# Androgen signaling restricts glutaminolysis to drive sex-specific Th17 metabolism in allergic airway inflammation

**DOI:** 10.1172/JCI177242

**Published:** 2024-10-15

**Authors:** Nowrin U. Chowdhury, Jacqueline-Yvonne Cephus, Emely Henriquez Pilier, Melissa M. Wolf, Matthew Z. Madden, Shelby N. Kuehnle, Kaitlin E. McKernan, Erin Q. Jennings, Emily N. Arner, Darren R. Heintzman, Channing Chi, Ayaka Sugiura, Matthew T. Stier, Kelsey Voss, Xiang Ye, Kennedi Scales, Evan S. Krystofiak, Vivek D. Gandhi, Robert D. Guzy, Katherine N. Cahill, Anne I. Sperling, R. Stokes Peebles, Jeffrey C. Rathmell, Dawn C. Newcomb

**Affiliations:** 1 Department of Pathology, Microbiology, and Immunology,; 2 Vanderbilt Center for Immunobiology, and; 3 Department of Medicine, Vanderbilt University Medical Center, Nashville, Tennessee, USA.; 4 Department of Cellular and Molecular Biology, Vanderbilt University, Nashville, Tennessee, USA.; 5 Department of Medicine, University of Wisconsin, Madison, Wisconsin, USA.; 6 Department of Medicine, University of Virginia, Charlottesville, Virginia, USA.

**Keywords:** Immunology, Pulmonology, Asthma, Sex hormones, T cells

## Abstract

Female individuals have an increased prevalence of many Th17 cell–mediated diseases, including asthma. Androgen signaling decreases Th17 cell–mediated airway inflammation, and Th17 cells rely on glutaminolysis. However, it remains unclear whether androgen receptor (AR) signaling modifies glutamine metabolism to suppress Th17 cell–mediated airway inflammation. We show that Th17 cells from male humans and mice had decreased glutaminolysis compared with female individuals, and that AR signaling attenuated Th17 cell mitochondrial respiration and glutaminolysis in mice. Using allergen-induced airway inflammation mouse models, we determined that females had a selective reliance upon glutaminolysis for Th17-mediated airway inflammation, and that AR signaling attenuated glutamine uptake in CD4^+^ T cells by reducing expression of glutamine transporters. In patients with asthma, circulating Th17 cells from men had minimal reliance upon glutamine uptake compared to Th17 cells from women. AR signaling thus attenuates glutaminolysis, demonstrating sex-specific metabolic regulation of Th17 cells with implications for Th17 or glutaminolysis targeted therapeutics.

## Abstract 2

Th17 cells require glutaminolysis. Androgen receptor signaling decreased Th17-mediated airway inflammation by limiting Th17 cell glutamine uptake, providing a mechanism for sex-specific Th17 effector function.

## Graphical abstract

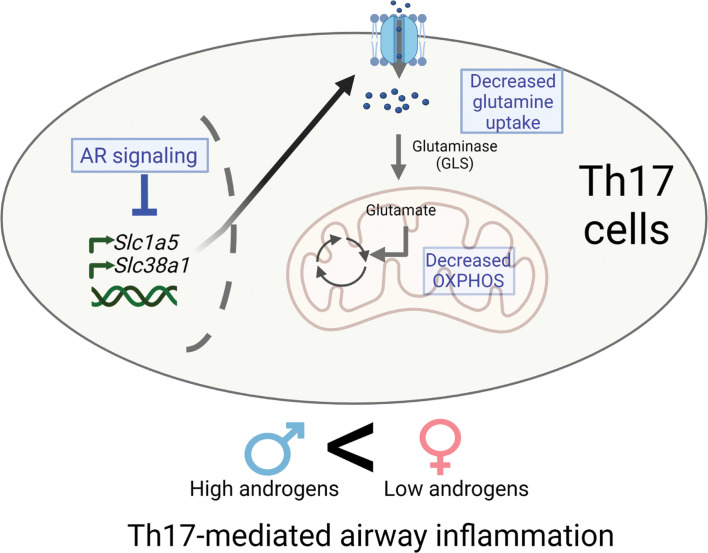

## Introduction

Cellular metabolism drives CD4^+^ T cell differentiation and stability, and CD4^+^ T cell metabolic pathways are promising therapeutic targets for cancers, autoimmune diseases, and asthma (1–3). Once activated, effector CD4^+^ T cells substantially increase glycolysis to proliferate and undergo clonal expansion (2, 4). However, the reliance on other metabolic pathways is required for maximal effector function and varies between the CD4^+^ T cell subsets of Th1, Th2, Th17, and regulatory T cells (Tregs) (3–6). Prior research focused on the metabolic pathways required for Th1, Th17, and Tregs, as these CD4^+^ T cell subsets are critical in pathogenesis of cancers and autoimmune diseases. Here, we focus on how androgen signaling through the androgen receptor (AR) alter metabolic pathways critical for Th17 and Th2 cells, cell types important in allergic airway inflammation and asthma.

Differential and overlapping metabolic pathways are essential for Th2 and Th17 cells. Th2 cells rely upon fatty acid oxidation and PPAR-γ signaling (7), and we recently showed that glutaminolysis was important for Th2 function during allergic airway inflammation (8). Th17 cells require de novo fatty acid synthesis (9) and one carbon metabolism (10) for differentiation, effector function, and cytokine expression (11). Further, glutaminolysis is essential for Th17 cell differentiation and effector function, and glutaminolysis may also inhibit Th1 cell differentiation (8, 11–13). Glutaminolysis involves the uptake and conversion of glutamine to glutamate by glutaminase (GLS). Glutamate can then be converted to α-ketoglutarate, which can enter the tricarboxylic acid (TCA) cycle, be converted to glutathione (GSH) for ROS management, modify chromatin methylation patterns, and/or be used for other metabolic processes (1, 14–16).

While metabolic needs for Th1, Th2, and Th17-mediated diseases have been examined, it remains unexplored how sex hormones modify these metabolic pathways. Understanding the role of sex hormones is critical since there is a female predominance in the prevalence and/or severity of many immune-mediated diseases, including asthma, multiple sclerosis, lupus, rheumatoid arthritis, and type I diabetes (17). This female sex bias has been attributed to a myriad of reasons, including the impact of sex hormones, X and Y chromosomal differences, and social and environmental factors (17, 18). Yet, the role of immunometabolism is unclear.

Prior research on sex hormone signaling and cell metabolism in cancer showed androgens signaling through the AR altered cell metabolism. In prostate cancer cells, AR signaling dysregulated glycolysis, the TCA cycle, amino acid metabolism, and fatty acid metabolism through transcriptional, epigenetic, and amino acid transport changes (19–21). Further, AR signaling increased glutamine metabolism and utilization in gliomas (22). However, cancer cells can have dysregulation of normal metabolic patterns and dysregulation of sex hormone receptor expression, and the impact of AR signaling on CD4^+^ T cell metabolism during inflammation is unknown. Various studies described the impact of AR signaling on immune cells and T cells through reductions in function or increased expression of exhaustion markers (23–26), but defining how sex hormones affect CD4^+^ T cell metabolism would provide a foundational mechanism for sex differences in CD4^+^ T cell subset effector function.

A previous study from our group showed that patients with asthma had increased numbers of CD4^+^ effector T cells and increased expression of metabolic enzymes in their CD4^+^ T cells compared with those of people in the healthy control group (8). However, the sex of participants and the effect of sex hormones on these cells was not considered. Given the importance of distinct metabolic programs on T cell function, we sought to determine the role of AR signaling in modifying CD4^+^ T helper cell metabolism in vitro using human and mouse cells and in vivo using a mouse model of allergen-induced airway inflammation. Here, we show that AR signaling directly decreased glutamine uptake and glutaminolysis in Th17 cells to reduce IL-17A–mediated inflammation.

## Results

### Metabolic markers in human CD4^+^ T cells from the mediastinal lymph nodes are decreased in men compared with women.

**Figure 1:**
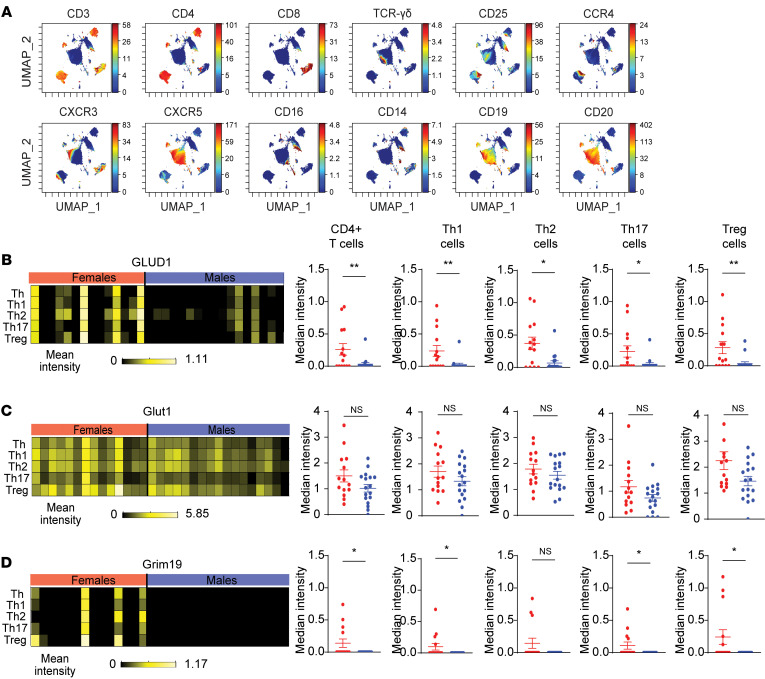
CyTOF of lung-draining lymph nodes of deceased donor patients reveals sex differences in T cells and metabolic protein expression. CyTOF was conducted on human lung draining lymph nodes from deidentified female and male deceased donors. (**A**) UMAP visualization of cell surface markers in all samples. (**B**–**D**) Median expression of metabolic markers on CD4^+^ T cells, Th1, Th2, Th17, and Treg cells. Gating strategies and population definitions are shown in Figure S1. Data are expressed as mean ± SEM: *n* = 14 females and *n* = 17 males *P < 0.05, **P < 0.01; 2-tailed Mann-Whitney *U* test. See Supplemental Figures 1 and 2 and Supplemental Tables 1 and 2.

Metabolic markers are differentially expressed based on the metabolic requirements of CD4^+^ T cells (8, 13, 27). Using excised mediastinal lymph nodes from deceased human male and female donors (ages 18–55) that did not qualify for lung transplant, we conducted mass cytometry (CyTOF) using an antibody panel focused on T cells and metabolic markers (Supplemental Table 1; supplemental material available online with this article; https://doi.org/10.1172/JCI177242DS1). Donors chosen were matched for age, race, and ethnicity (Supplemental Table 2). Surface expression of chemokine receptors, CCR4, and CXCR3, as well as other surface markers were used to identify CD4^+^ T cells and subset these cells into Th1, Th2, Treg, and Th17 cells as previously described in PBMCs and lung-draining lymph nodes (Figure 1A) (28, 29). Gating strategies and CD4^+^ T cell population definitions are shown in Supplemental Figure 1. The median expression of metabolic markers was also determined in CD4^+^ T cell subsets (Figures 1, B–D and Supplemental Figure 2). Metabolic markers included Glut1 for glycolysis, GLUD1 for glutaminolysis, Cpt1a for fatty acid metabolism, and Grim19, ATP5a, and cytochrome C as various parts of the electron transport chain in mitochondrial metabolism. The CD4^+^ T cells and all subsets of Th1, Th2, Th17, and Tregs had decreased expression in GLUD1 in male compared with female individuals (Figure 1B). Additionally, Grim19 was decreased in CD4^+^ T cells, Th17, and Tregs in males compared with females, and ATP5a was decreased in Tregs from male compared with female individuals (Figures 1D and Supplemental Figure 2B). Other metabolic markers, Glut1, Cpt1a, and cytochrome C, were not different between the male and female CD4^+^ T cell subsets that were examined (Figures 1C and Supplemental Figure 2, A and C). These data show that effector CD4^+^ T cells from human lung draining lymph nodes from males are metabolically distinct from T cells from females, with decreased expression of GLUD1 and Grim19.

### AR signaling intrinsically decreases Th17- but not Th2-mediated inflammation.

**Figure 2:**
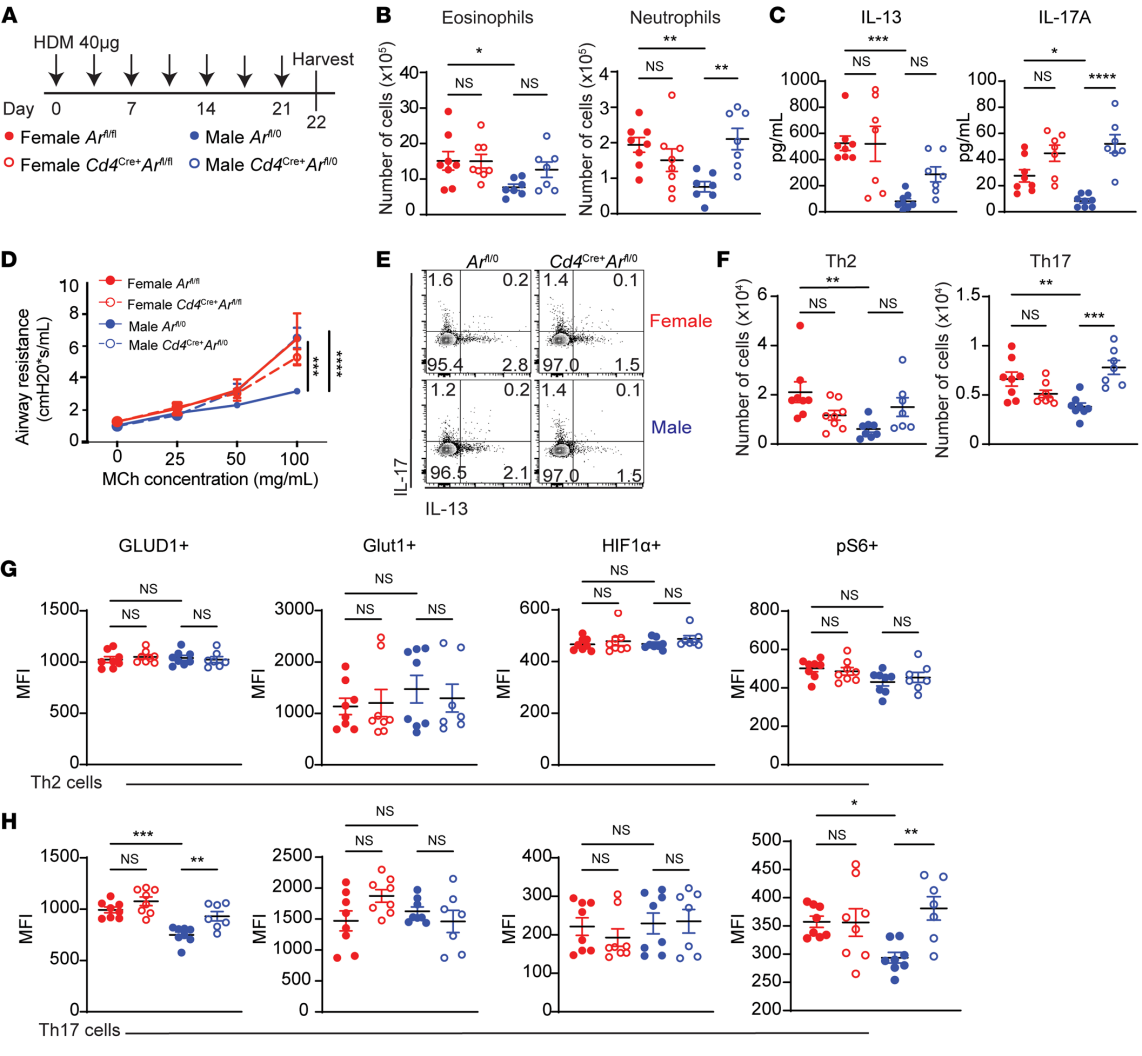
AR signaling in CD4^+^ T cells reduces Th17-driven neutrophilic inflammation during airway inflammation. (**A**) Model of HDM allergen challenge. Female and male *Ar^fl^ and Cd4^Cre+^ Ar ^fl^* mice were challenged intranasally with 40 μg of HDM 3 times per week for 3 weeks. BAL fluid and lungs were harvested 24 hours after the last challenge. (**B**) Quantification of eosinophils and neutrophils in the BAL fluid from mice after HDM challenge. (**C**) IL-13 and IL-17A protein expression in BAL fluid after HDM challenge. (**D**) Airway hyperresponsiveness as measured by FlexiVent with increasing doses of methacholine was also conducted on HDM-challenged female and male *Ar*^fl^ and *Cd4*^Cre+^ *Ar*^fl^ mice 48 hours after last challenge (Data show mean ± SEM, *n* = 4–6 mice per group). (**E**) Representative flow diagrams of IL-13^+^ Th2 cells and IL-17A^+^ Th17 cells. (**F**) Quantification of lung Th2 and Th17 cells after HDM challenge (**G** and **H**) Expression of GLUD1, Glut1, HIF1-α, and pS6 in lung Th2 cells (**G**) and Th17 cells (**H**) after HDM challenge. (**B**, **C**, and **E**–**H**) Data are expressed as mean ± SEM: *n* = 7–9 mice per group combined from 2 independent experiments. *P < 0.05, **P < 0.01, ***P < 0.001, ****P < 0.0001; ANOVA with Tukey’s post hoc test. See Supplemental Figures 3, 4, and 5.

Reports from our group and others showed that glutaminolysis increased airway inflammation and IL-17A production from Th17 cells (8) and that AR signaling decreased type 2 and nontype 2 inflammation during allergic airway inflammation (25, 26, 30). Specifically, we previously showed a cell-intrinsic role of AR signaling in Th17 cell–driven inflammation in a house dust mite (HDM) model through bone marrow chimera experiments using T cells from WT male and *Ar^Tfm^* male mice (25). Based on these findings, we next determined if AR signaling in CD4^+^ T cells attenuated HDM-induced airway inflammation and modified expression of metabolites in CD4^+^ T cells. As shown in Figure 2A, *Ar^fl/fl^* female*, Cd4^Cre+^ Ar^fl/fl^* female, *Ar*^fl/0^ male, and *Cd4^Cre+^ Ar^fl/0^* male mice were challenged with 40 μg of HDM 3 times a week for 3 weeks. Lungs and bronchoalveolar lavage (BAL) fluid were harvested 1 day after the last challenge to assess airway inflammation. HDM-challenged *Ar^fl/fl^* female mice had increased numbers of eosinophils and neutrophils in the BAL fluid compared with *Ar^fl/0^* male mice, and *Cd4^Cre+^ Ar^fl/0^* male mice had increased neutrophil infiltration and no difference in eosinophils compared with *Ar^fl/0^* male mice (Figure 2B). IL-13 and IL-17 cytokine levels in BAL fluid were also significantly increased in *Ar^fl/fl^* female mice compared *Ar^fl/0^* male mice, and *Cd4^Cre+^ Ar^fl/0^* male mice had increased IL-13 and IL-17 levels compared with *Ar^fl/0^* male mice (Figure 2C). To determine if AR signaling in CD4^+^ T cells attenuated allergen-induced airway hyperresponsiveness (AHR) to methacholine, a physiological hallmark of asthma, we measured airway resistance in response to increasing concentrations of nebulized methacholine via Flexivent. Similar to what we have previously reported (25, 26), *Ar^fl/fl^* female mice had increased AHR compared with *Ar^fl/0^* male mice (Figure 2D, solid red versus solid blue lines). *Cd4^Cre+^ Ar^fl/0^* male mice had similar AHR compared with *Ar^fl/fl^* female mice (dotted blue line overlaid with solid red line) and no significant difference was determined between *Cd4^Cre+^ Ar^fl/fl^* female and *Ar^fl/fl^* female mice (dotted and solid red lines). These data show that AR signaling in CD4^+^ T cells attenuate HDM-induced airway inflammation and AHR.

Next, we conducted flow cytometry to determine the number of lung Th2, Th17, and Treg cells (Supplemental Figure 3A). HDM-challenged *Ar^fl/fl^* female mice also had increased numbers of lung Th2 and Th17 cells compared with *Ar^fl/0^* male mice, and removing AR signaling in the CD4 cells in the *Cd4^Cre+^ Ar^fl/0^* male mice increased numbers of lung Th17 cells (Figure 2, E and F). However, no differences in lung Th2 cells were observed in the HDM-challenged *Cd4^Cre+^ Ar^fl/0^* male mice compared with the *Ar^fl/0^* male mice (Figure 2, E and F). The lack of difference between eosinophils and lung Th2 cells in *Ar^fl/0^* male mice and *Cd4^Cre+^ Ar^fl/0^* were consistent with our prior studies using an in vivo adoptive transfer model showing that AR signaling had no direct effect on Th2 cells (25). In this study, we also showed AR signaling on all CD4-expressing cells did not attenuate the total number of lung Treg cells, but did decrease the ratio of lung Tregs to Th2 and Th17 cells (Supplemental Figure 3C). This confirms our previous findings using mice with AR deficiency only in Tregs (26).

Using flow cytometry, we also determined expression levels of the metabolic markers GLUD1, Glut1, HIF1α, and phospho-S6 (p-S6) in Th2 and Th17 cells (Figure 2, G and H and Supplemental Figure 3B). p-S6 is a marker for mTORC1 activation, a key regulator in T cell activation and metabolism. HIF1-α is a key metabolic regulator for Th17 cells (31). In Th2 cells, no differences were noted in the mean fluorescent intensity (MFI) of metabolic markers between any groups (Figure 2G). In Th17 cells, the MFI of GLUD1 and p-S6 were increased in *Cd4^Cre+^ Ar^fl/0^* males and *Ar^fl/fl^* females compared with *Ar*^fl/0^ male mice with no differences detected in Glut1 and HIF1-α (Figure 2H). Together, these data suggest that AR signaling intrinsically decreased allergen-induced lung Th17 cells and decreased GLUD1 expression, a marker of glutaminolysis, and p-S6 expression, a proxy for mTOR activation, in Th17 cells.

### Antagonism of the AR via flutamide increases Th17-mediated airway inflammation and expression of glutaminolysis-related enzymes.

To determine whether a pharmacological inhibitor of AR, flutamide, also increased Th17-mediated airway inflammation, we implanted slow-release flutamide or vehicle pellets (50 mg) s.c. in WT male mice. After 3 weeks, HDM was intranasally administered as outlined in Figure 2A. Administration of flutamide to WT male mice increased neutrophils in the BAL fluid, lung Th2 cells, and lung Th17 cells compared with vehicle-treated WT males (Supplemental Figure 4, A–C). These increases in neutrophils, lung Th2 cells, and lung Th17 cells were similar to WT female mice. No differences in eosinophils in the BAL fluid were determined in WT male mice administered vehicle or flutamide. Further, flutamide-treated WT male mice and WT female mice had increased GLUD1 and pS6 expression in Th17 cells, but not Th2 cells, compared with vehicle-treated WT male mice (Supplemental Figure 4, D and E). These data show that pharmacological inhibition of AR signaling with flutamide increased Th17-mediated neutrophilic airway inflammation and expression of glutaminolysis-related enzymes in Th17 cells.

### Estrogen receptor α signaling in CD4^+^ T cells increases neutrophil infiltration and Th17 cells in the lung, but does not impact the expression of metabolic enzymes in Th17 cells.

Our data and others have shown that female individuals have increased allergen-induced airway inflammation compared with male individuals, and that estrogen signaling through estrogen receptor α (ER-α) increased this inflammation (25, 32, 33). Therefore, we also wanted to determine if ER-α signaling specifically in CD4^+^ cells increased HDM-induced neutrophil infiltration into BAL fluid, numbers of lung Th17 cells, and GLUD1 and pS6 expression in Th17 cells by using our HDM protocol in male and female *Cd4^Cre+^ Esr1^fl/fl^* and *Esr1^fl/fl^* mice. *Cd4^Cre+^ Esr1^fl/fl^* female mice had decreased neutrophils in BAL fluid, IL-17A production in whole-lung homogenates, and numbers of lung Th17 cells compared with *Esr1^fl/fl^* female mice (Supplemental Figure 5, A–D). No differences in eosinophils in the BAL fluid, IL-13 production in whole lung homogenates, or numbers of lung Th2 cells were observed between *Cd4^Cre+^ Esr1^fl/fl^* and *Esr1^fl/fl^* female mice (Supplemental Figure 5, A and B). Additionally, no differences in the numbers of Th2 and Th17 cells or in expression of metabolic markers were determined between *Cd4^Cre+^ Esr1^fl/fl^* and *Esr1^fl/fl^* female mice (Supplemental Figure 5, C–F). Interestingly, male *Cd4^Cre+^ Esr1^fl/fl^* had decreased GLUD1 and pS6 expression in Th2 cells compared with male *Esr1^fl/fl^* mice. The reason for this difference is unclear and warrants future investigation. These data show that ER-α signaling in CD4^+^ T cells increased IL-17A production and neutrophil infiltration into the airway but that ER-α signaling in CD4^+^ T cells does not affect HDM-induced GLUD1 and p-S6 in lung Th17 cells.

### AR signaling reduces mitochondrial metabolism in differentiated Th17 cells.

**Figure 3:**
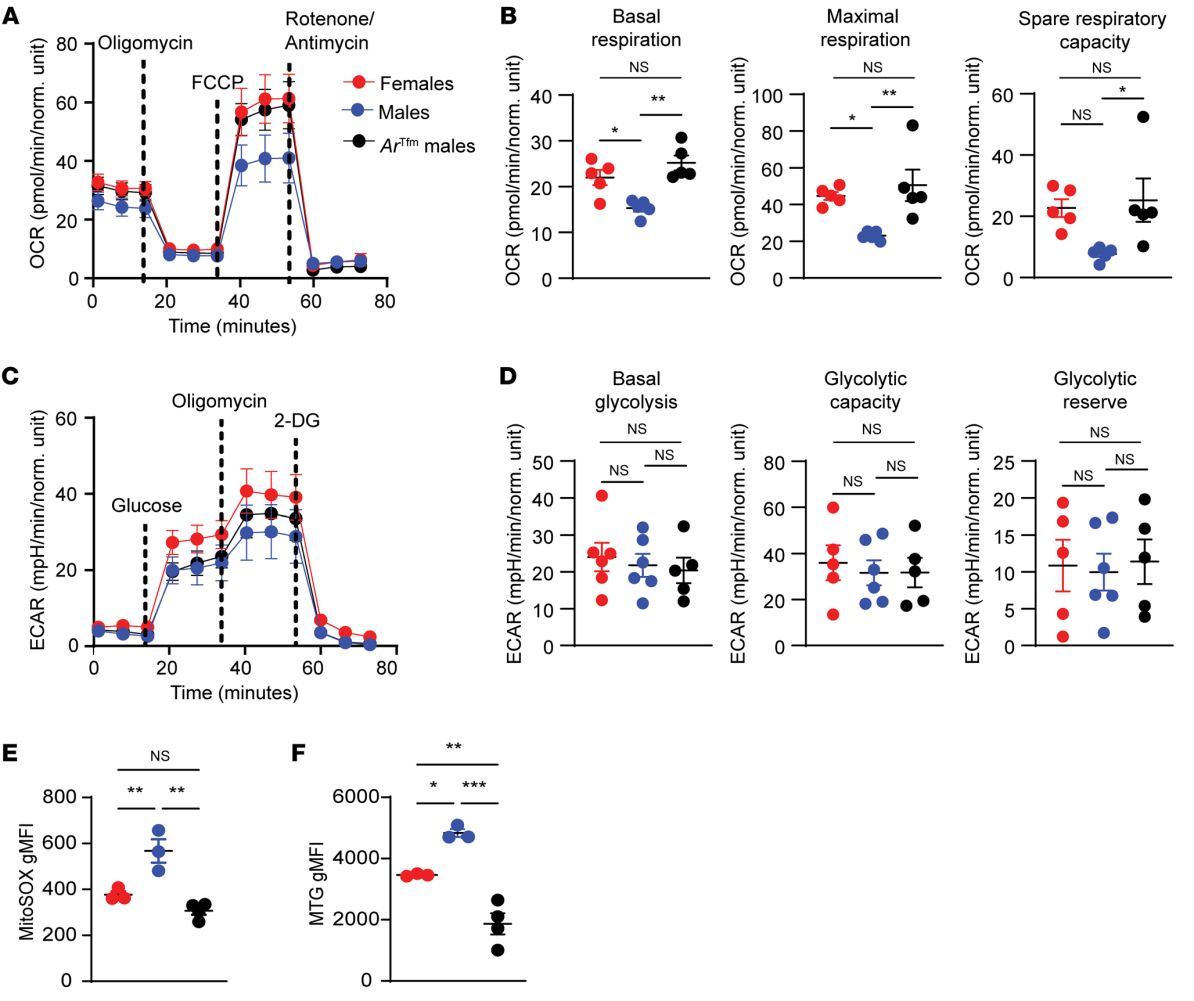
AR signaling modifies mitochondrial metabolism but not glycolysis in Th17 cells. (**A**) Seahorse MitoStress Test on differentiated Th17 cells from WT female, WT male, and *Ar^Tfm^* male mice to measure mitochondrial respiration using oxygen consumption rate (OCR) (*n* = 5 mice per group from 2 independent experiments). (**B**) Quantified measures of basal respiration, maximal respiration, and spare respiratory capacity from panel **A**. (**C**) Seahorse GlycoStress test on Th17 cells from WT female, WT male, and *Ar^Tfm^* male mice to measure glycolysis using extracellular acidification rate (ECAR) (*n* = 5–6 mice per group from 2 independent experiments). (**D**) Quantified measures of basal glycolysis, glycolytic capacity, and glycolytic reserve from panel **C**. (**E** and **F**) Expression of MitoSOX Red, a mitochondrial superoxide marker, and Mitotracker green (MTG), a stain to measure mitochondrial mass, in differentiated Th17 cells from WT female, WT male, and *Ar^Tfm^* male mice (*n* = 3–4 mice per group, representative of 2 independent experiments). Data show mean ± SEM, *P < 0.05, **P < 0.01; ANOVA with Tukey’s post hoc test. See Supplemental Figure 6 and 7.

Given our findings that AR signaling decreased metabolic markers in Th17 cells in mice, we next determined if AR signaling functionally decreases Th17 and Th2 metabolism in vitro. Naive splenic CD4^+^ T cells from WT male, WT female, and *Ar*^Tfm^ male mice were differentiated into Th2 and Th17 cells. *Ar*^Tfm^ mice have a global mutation in the AR making it nonfunctional. Similar to our earlier findings, we confirmed that differentiated Th17 cells from WT female mice and *Ar*^Tfm^ male mice had increased expression of IL-17A production in culture supernatants and increased numbers of Th17 differentiated cells, defined as ROR-γT^+^ CD4^+^ T cells, compared with WT male mice (Supplemental Figure 6A), showing that AR signaling limits Th17 differentiation in culture (25). Next, we next determined mitochondrial respiration and glycolysis using extracellular flux analysis (34). Th17 cells from WT female and *Ar*^Tfm^ male mice had increased basal respiration, maximal respiration, and spare respiratory capacity compared with Th17 cells from WT male mice (Figure 3, A and B). A similar experiment was conducted using Th17 cells from WT female, WT male, and *Ar*^Tfm^ male mice differentiated using HPLM media and similar results were observed as in Figure 3, A and B (data not shown). Consistent with our in vivo allergen model, AR signaling had no direct effects on Th2 mitochondrial respiration in vitro (Supplemental Figure 7, A and B). AR signaling did not significantly impact basal glycolysis, glycolytic capacity, or glycolytic reserve in Th17 or Th2 cells (Figure 3, C and D and Supplemental Figure 7, C and D, respectively).

To further characterize the impact of AR signaling on Th17 and Th2 cell mitochondria and understand whether AR signaling modified the generation of ROS, we measured MitoSox Red via flow cytometry. ROS suppress Th17 differentiation and effector function (6, 35, 36). Th17 cells from *Ar*^Tfm^ male mice and female mice had decreased mitochondrial superoxide production compared with Th17 cells from WT male mice (Figure 3E). We further measured the role of AR signaling in mitochondrial mass using MitoTracker Green (MTG) staining via flow cytometry. Th17 cells from *Ar*^Tfm^ male mice and female mice also had decreased mitochondrial mass compared with those from WT male mic,e as detected by MitoTracker Green (Figure 3F). There were no differences in MitoSox Red or MitoTracker Green staining in Th2 cells (Supplemental Figure 7, E and F). Next, we determined if AR signaling modified mitochondrial morphology by transmission electron microscopy in differentiated Th17 cells. Mitochondria from *Ar*^Tfm^ male mice had decreased area compared with those from WT males, but no there were no differences in aspect ratio (a measure of mitochondrial roundness) or internal density (Supplemental Figure 7, G and H). Collectively, these data show AR signaling specifically restricts oxidative metabolic output in Th17 cells, while increasing mitochondrial mass and area.

### AR signaling reduces glutamine metabolism in differentiated Th17 cells.

**Figure 4:**
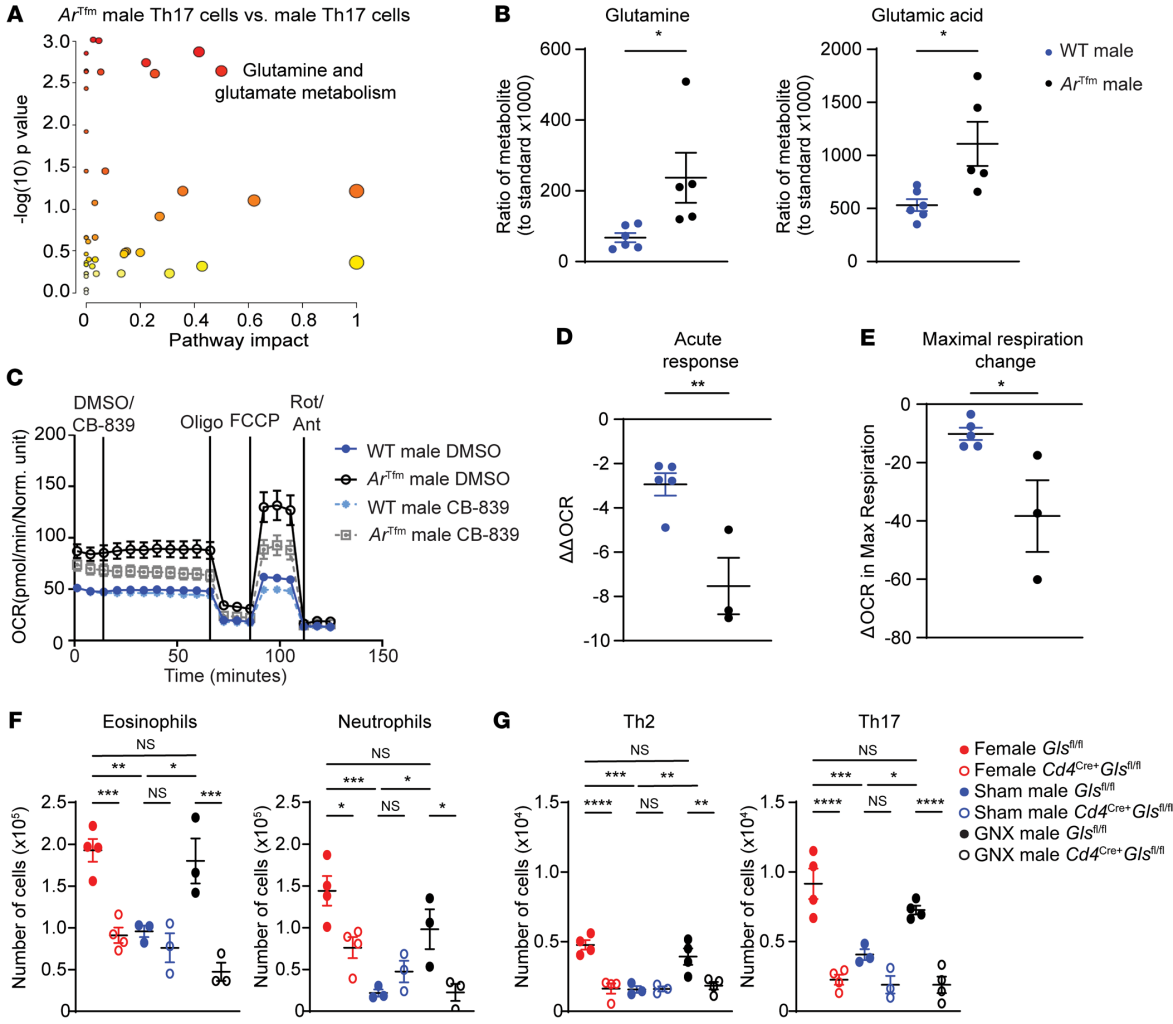
AR signaling reduces glutamine metabolism in Th17 cells. (**A**) Pathway analysis on targeted metabolomics from differentiated Th17 cells from WT male and *Ar^Tfm^* male mice (*n* = 5–6 mice per group from 2 independent experiments, statistical analysis by MetaboAnalyst Global test, larger circle indicates larger impact, darker shade indicates increased significance). (**B**) Intracellular glutamine and glutamate levels from differentiated Th17 cells from WT male and *Ar^Tfm^* male mice measured by mass spectrometry (Data show mean ± SEM, *n* = 5–6 mice per group). (**C**) Seahorse Substrate Oxidation Assay using CB-839, an inhibitor of glutaminase, on differentiated Th17 cells from WT male and *Ar^Tfm^* male mice to measure dependance of mitochondrial respiration of Th17 cells on glutamine metabolism. (**D**) Quantified ΔΔOCR from **C**. ΔOCR was calculated by determining the difference in measured OCR after DMSO or CB-839 injection. ΔΔOCR was calculated as follows: ΔOCR_CB-839_ _basal_ – ΔOCR_DMSO_ _basal_. See Supplemental Figure 5A (**E**) Quantified from **C** and calculated as OCR_CB-839_ _Max_ – OCR_DMSO_ _Max_. *P < 0.05, unpaired 2-tailed *t* test. See Supplemental Figure 5 and Supplemental Table 3. (**F** and **G**) HDM-induced airway inflammation in female and sham or gonadectomized (GNX) male *Gls*^fl/fl^ and *Cd4*^Cre+^ *Gls*^fl/fl^ mice were sensitized and challenged with HDM as described in Figure 2A. A day following the last challenge, BAL fluid and lungs were harvested. (**F**) Eosinophils and neutrophils in BAL fluid and (**G**) Lung Th2 and Th17 cells quantified by flow cytometry. Data show mean ± SEM, *n* = 3–4 mice per group, *P < 0.05, ** *P* < 0.01, *** *P* < 0.001, **** *P* < 0.0001; ANOVA with Tukey’s post hoc test. See Supplemental Figure 8 and 9 and Supplemental Table 3.

Since AR signaling decreased mitochondrial metabolism in Th17 cells, we next conducted targeted metabolomics and pathway analysis on differentiated Th17 cells from WT male and *Ar*^Tfm^ male mice (37). Glutamine metabolic pathways were significantly increased in Th17 cells from *Ar*^Tfm^ male mice compared with Th17 cells from WT males (Figure 4A and Supplemental Table 3). Th17 cells from *Ar*^Tfm^ male mice also had increased glutamine and glutamate compared with Th17 cells from WT males (Figure 4B).

We next wanted to determine the contribution of glutaminolysis to oxidative phosphorylation in Th17 cells from WT and *Ar*^Tfm^ male mice by conducting a Seahorse Substrate Oxidation Assay using CB-839, an inhibitor of GLS (38). To conduct this assay, basal respiration was determined during the first 10 minutes of the assay. CB-839 (10 μM) or vehicle control (DMSO) was then injected onto Th17 cells (at 10 minutes) and incubated for 60 minutes prior to starting a standard Seahorse MitoStress assay. As shown graphically in Supplemental Figure 8, ΔOCR_basal_ was determined by subtracting the basal OCR measurement at the end of the CB839 incubation period from the basal OCR at the time of CB-839 or vehicle injection. ΔΔOCR_basal_ was calculated as ΔOCR_CB-839 basal_ – ΔOCR_DMSO_ _basal_, providing a measure of how reliant the cells were on glutaminolysis (Supplemental Figure 8). Th17 cells from *Ar*^Tfm^ male mice had a greater decrease in ΔΔOCR_basal_ and decreased ΔOCR_max_ compared with Th17 cells from WT male mice (Figure 4, C–E). These results show that AR signaling decreased glutaminolysis-dependent oxidative phosphorylation in Th17 cells, providing a mechanism for AR-dependent decreases in Th17 differentiation and function.

### Disruption of glutaminolysis restricts airway inflammation in low androgen environments.

We next explored if androgens attenuated the reliance on glutaminolysis in lung Th17 cells in vivo. *Cd4^Cre+^ Gls^fl/fl^* and *Gls^fl/fl^* male mice underwent sham or gonadectomy (GNX) surgery at 4 weeks of age, prior to puberty for males. At 8 weeks of age, sham and GNX *Cd4^Cre+^ Gls^fl/fl^* and *Gls^fl/fl^* male mice as well as *Cd4^Cre+^ Gls^fl/fl^* and *Gls^fl/fl^* female mice underwent the HDM protocol outlined in Figure 2A. GNX males and female *Cd4^Cre+^ Gls^fl/fl^* mice had decreased eosinophils and neutrophils in the BAL fluid and decreased lung Th2 and Th17 cells compared with GNX males or female *Gls^fl/fl^* mice, respectively (Figure 4F). No differences in any of these endpoints were determined between HDM-challenged *Cd4^Cre+^ Gls^fl/fl^* and *Gls^fl/fl^* male mice. Further, *Cd4^Cre+^ Gls^fl/fl^* female mice had decreased AHR to methacholine, a physiological hallmark of asthma, compared with *Gls^fl/fl^* female mice (Supplemental Figure 9). This change in AHR was not detected in HDM-challenged *Cd4^Cre+^ Gls^fl/fl^* and *Gls^fl/fl^* male mice. These findings show that Th2 and Th17 cells from males with high AR signaling do not rely on glutaminolysis during allergic airway inflammation. Depletion of AR signaling through GNX restored this reliance upon glutaminolysis in Th2 and Th17 cells of HDM-challenged male mice. Further, these findings show that glutaminolysis in Th2 and Th17 cells is required for maximal HDM-induced airway inflammation, providing an in vivo mechanism for how AR signaling attenuates HDM-induced airway inflammation.

### AR signaling reduces glutamine uptake in Th17 cells.

**Figure 5:**
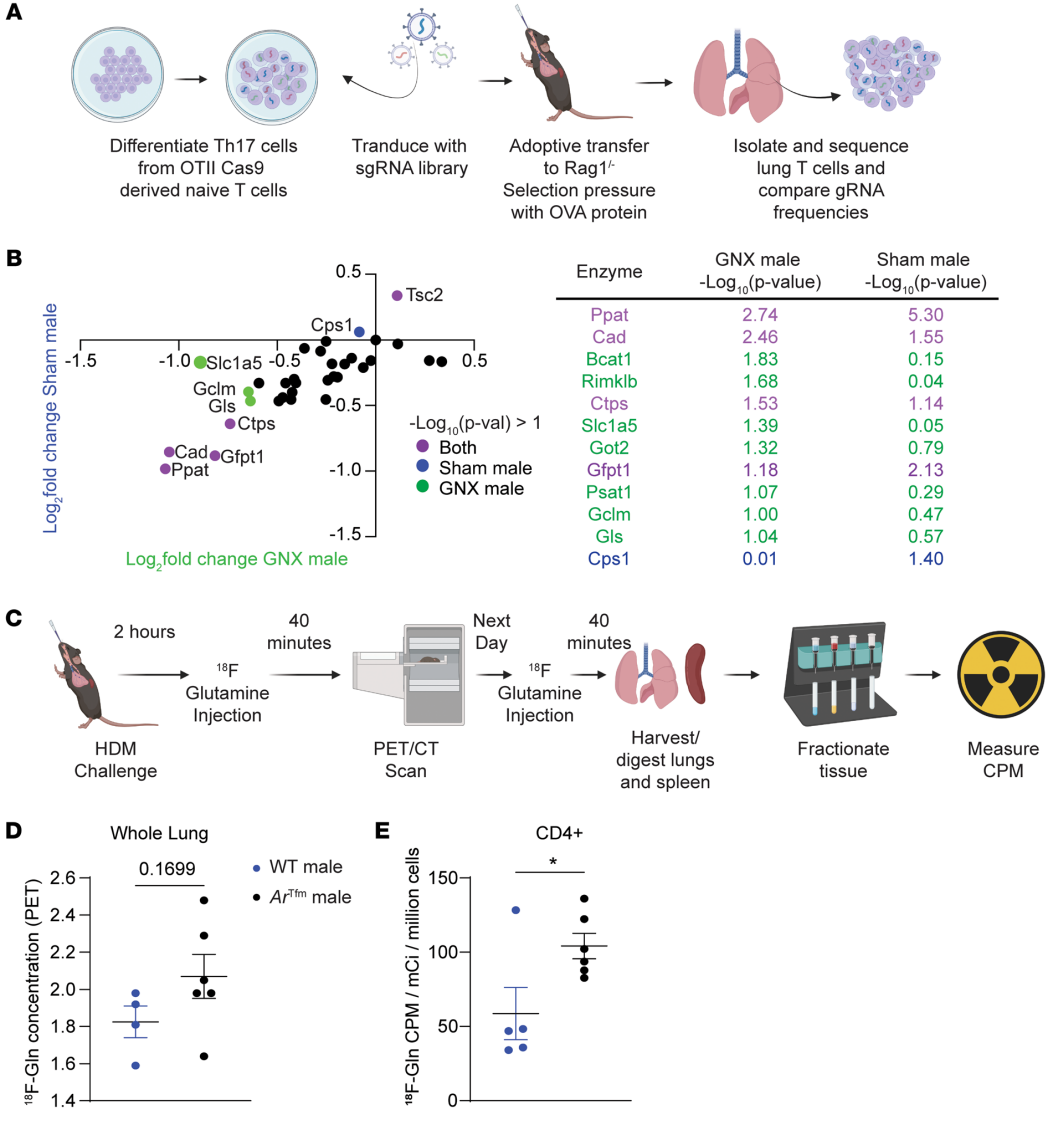
AR signaling reduces glutamine uptake in T cells. (**A**) Model of targeted CRISPR screen using a glutamine library on differentiated Th17 cells from male and female OT-II Cas9 mice in an OVA-induced lung inflammation model. (**B**) Change in gRNA abundance in lung Th17 cells from male mice (y-axis) and GNX male mice (x-axis) with table showing statistics (Data show mean ± SEM, *n* = 4–5 mice, statistical analysis by MAGeCK affected represented by color of dot as shown in legend). (**C**) Model of F18-Glutamine PET studies in HDM-induced lung inflammation as shown in Figure 2A. (**D**) Quantification of ^18^F-Glutamine concentration in whole lung by PET/CT imaging (*n* = 4–6 mice per group). (**E**) Quantification of ^18^F-Glutamine concentration in lung CD4^+^ T cells by magnetic separation and γ counting, normalized to viable cells (Data show mean ± SEM, *n* = 4–6 mice per group). ***P < 0.001, unpaired 2-tailed *t* test. See Supplemental Figure 10 and 11.

Based on our in vitro and in vivo findings of AR signaling differentially impacting glutaminolysis reliance, we conducted in vivo glutamine metabolism-targeted CRISPR screens. We used OVA-specific Th17-differentiated cells from OT-II Cas9 from GNX males and sham-operated males (Figure 5) or male and female mice (Supplemental Figure 10) to test which specific genes in the pathway were responsible for the sex-specific differences in glutamine reliance (10). Similar to GNX experiments in Figure 4F, mice underwent sham or GNX surgery at 4 weeks of age, prior to puberty for males. At 8–10 weeks of age, Th17 differentiated cells from OT-II Cas9 GNX and sham-operated male mice were transduced with gRNAs from a glutamine CRISPR library and then adoptively transferred into *Rag1*^–/–^ male mice (see diagram in Figure 5A). gRNAs from Th17 cells were also isolated prior to adoptive transfer to provide a baseline comparison. *Rag1*^–/–^-recipient mice were administered OVA protein intranasally to establish OVA-induced airway inflammation. Lungs were harvested 1 day following the last OVA challenge and OVA-specific T cells were isolated by cell sorting. The gRNA for TSC2 was enriched in both GNX and sham-operated males, supporting an inhibitory role in Th17 cells. The gRNAs for *Ppat* and *Cad* were depleted in both GNX and sham-operated males supporting that these genes increase Th17 cell proliferation. *Slc1a5*, a gene that encodes for the glutamine transporter ASCT2, and *Gls,* an enzyme that converts glutamine to glutamate, were significantly depleted only in GNX males and required for Th17 cell proliferation (Figure 5B). In a separate study, Th17-differentiated cells from OT-II Cas9 female and male mice were used in the same in vivo CRISPR screen and *Slc38a1*, a gene that encodes for another glutamine transporter (SNAT1), was significantly depleted in females and required for Th17 cell fitness (Supplemental Figure 10). Collectively, these data show that AR signaling attenuates glutamine uptake genes in Th17 cells during ongoing airway inflammation.

Next, we wanted to directly test if AR signaling decreased glutamine uptake in CD4^+^ T cells during ongoing allergic airway inflammation. We conducted an in vivo ^18^F-labelled glutamine uptake assay. As shown in the model in Figure 5C, we used ^18^F-glutamine tracing and magnetic cell separation to determine glutamine uptake in various cell types, as previously shown (39). ^18^F-glutamine levels were not significantly different in the whole lung by whole body PET/CT scan (Figure 5D and Supplemental Figure 11A), but glutamine uptake by CD4^+^ cells in the lungs was increased in *Ar*^Tfm^ male mice compared with the WT male mice (Figure 5E). Glutamine uptake was not different in CD4^+^ cells or CD4^–^ cells in *Ar*^Tfm^ male mice compared with the WT male mice in the spleen (Supplemental Figure 11B), showing tissue localized changes in CD4^+^ cell metabolism with intranasal HDM challenge. Interestingly, HDM-challenged *Ar*^Tfm^ male mice also had increased glutamine uptake in other cells, including CD11b^–^CD4^–^ lung cells compared with WT male mice, indicating effects of AR signaling on glutaminolysis in other cell types in the lung (Supplemental Figure 11C). In all, AR signaling reduced glutamine uptake in lung CD4^+^ cells during allergic airway inflammation, supporting a mechanism for decreased reliance of glutaminolysis in male Th17 cells.

### AR signaling decreases glutaminolysis and flux through the TCA cycle.

**Figure 6:**
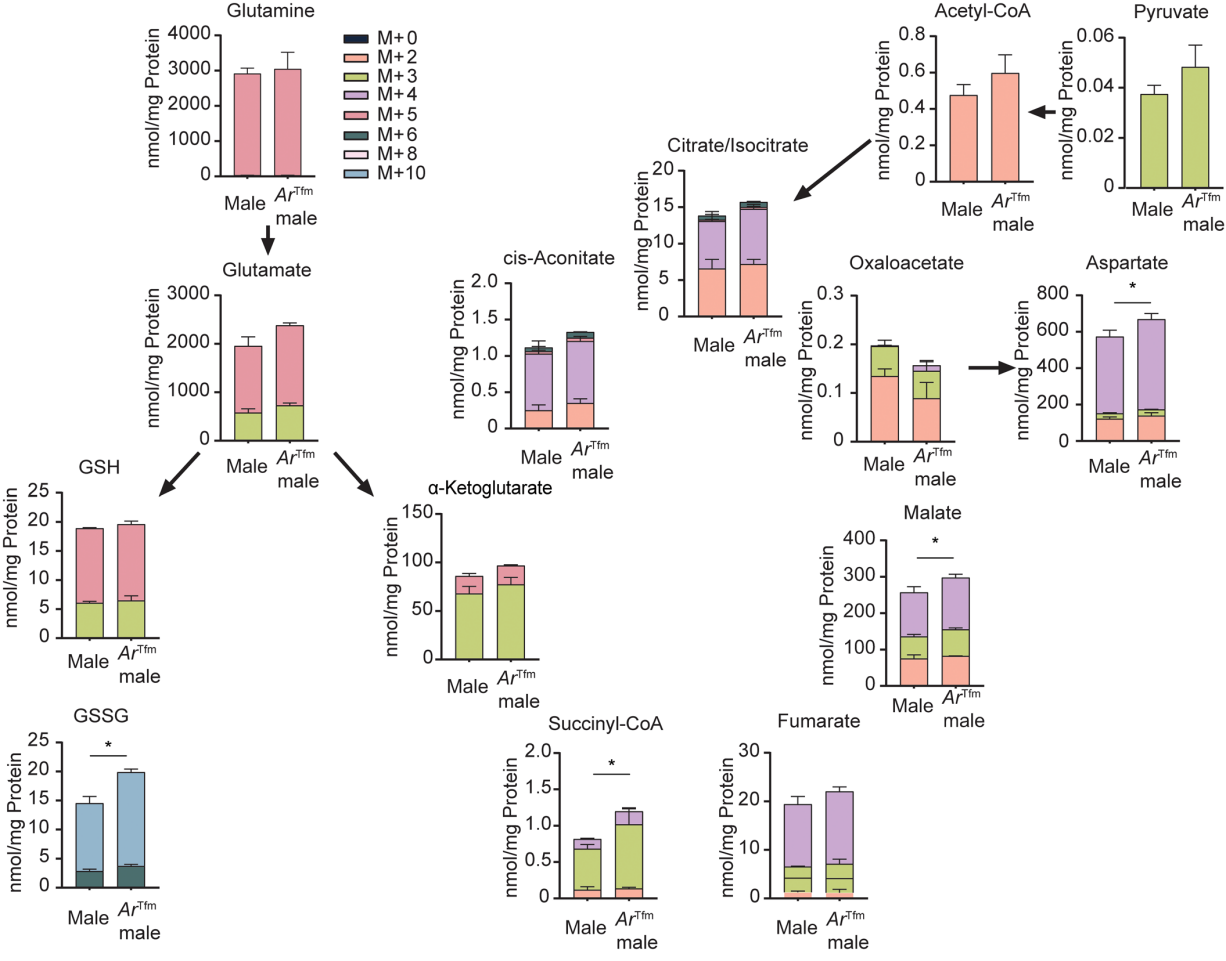
AR signaling reduces glutamine uptake and TCA cycle intermediates. Th17 cells were differentiated from WT male and *Ar*^Tfm^ male mice and pulsed with ^13^C_5_-glutamine for 4 hours. ^13^C-labelled metabolites were measured by liquid chromatography-tandem mass spectrometry and normalized to total protein within the sample. Colored bars indicate the number of carbons labelled on each isotopologue for each metabolite. Data show mean ± SEM, *n* = 3–5 samples per group, *P < 0.05, Welch’s 2-tailed *t* test.

To further delineate the significance of glutamine and its metabolic fate in Th17 cells, we utilized uniformly labelled ^13^C-glutamine tracing to determine the utilization of glutamine in differentiated Th17 cells from WT male and *Ar*^Tfm^ male mice (Figure 6). AR signaling decreased glutamate and the flux of glutamine through TCA intermediates such as succinyl-CoA and malate. Further, there may also be increased utilization of glutamine in the malate/aspartate shuttle, with tracing showing that *Ar*^Tfm^ male mice have increased malate and asparatate levels. Most strikingly, AR signaling reduces labelled glutamine conversion from reduced GSH to oxidized glutathione (GSSG), with Th17 cells from ArTfm male mice having increased GSSG compared with Th17 cells from WT male mice. GSSG is important in the management of ROS, and these data are consistent with previous studies showing that increased ROS decreased Th17 differentiation (12). In all, in vitro AR signaling decreases glutamine utilization for GSSG as well as TCA flux during glutaminolysis.

### AR signaling decreases accessibility to genes related to glutamine uptake.

**Figure 7:**
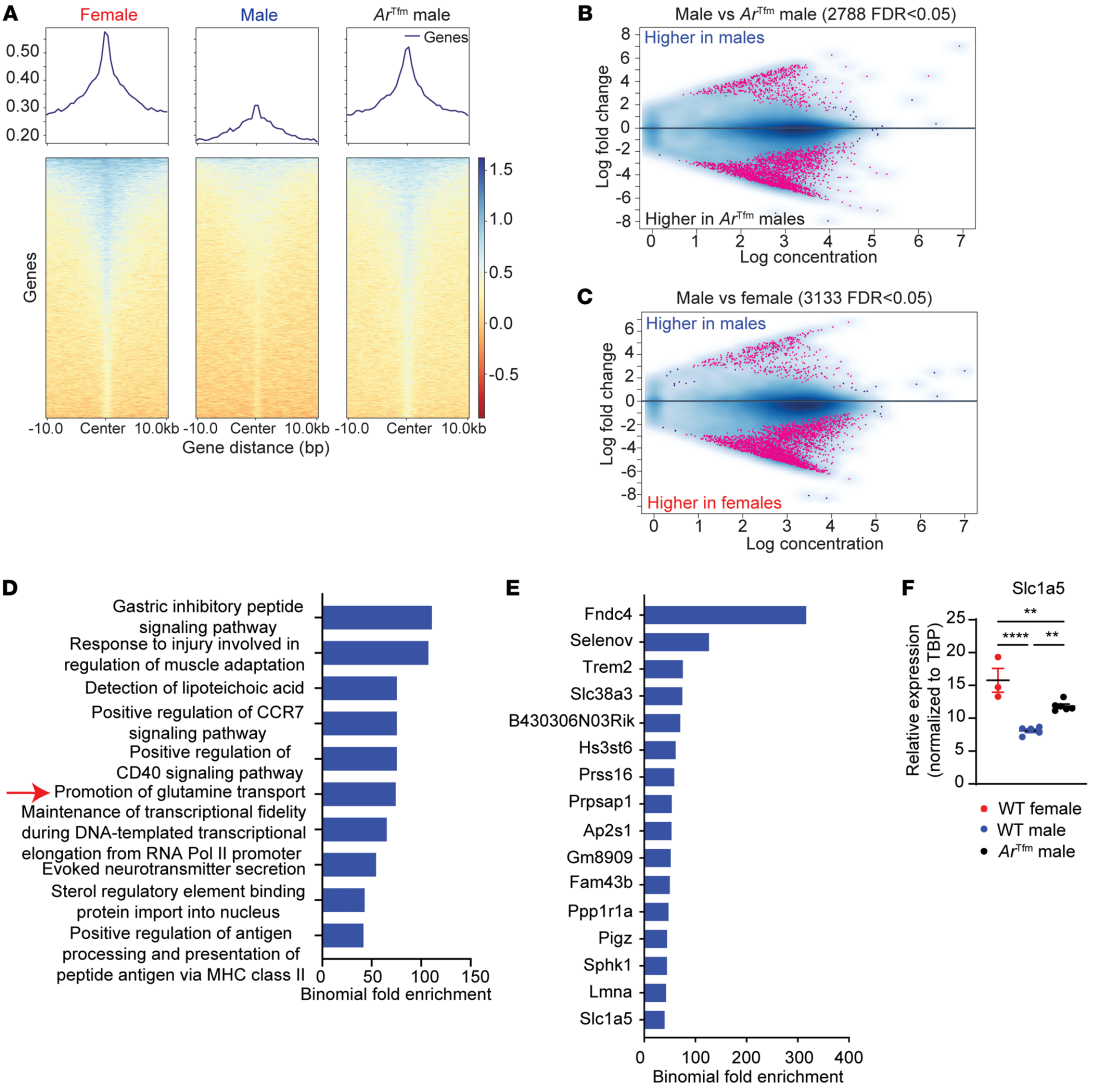
AR signaling alters H3K27 trimethylation in Th17 cells. CUT & RUN analysis for H3K27me3 was conducted on Th17 cells differentiated from WT male, WT female, and *Ar*^Tfm^ male mice (*n* = 3 per group). (**A**) Heatmaps of H3K27me3 to IgG control ratio in ± 10 kb regions around promoters of UCSC known genes in Th17 cells measured by CUT&RUN. (**B** and **C**) Analysis of differentially methylated areas in Th17 cells from male and *Ar*^Tfm^ male mice (panel **B**) or Th17 cells from males and females (panel **C**). (**D**) GREAT analysis to find differences in gene ontology pathways in Th17 cells from WT male versus *Ar*^Tfm^ male mice. (**E**) GREAT analysis to find differences in H3K27me3 tagged genomic regions in Th17 cells from WT male versus *Ar^Tfm^* male mice. (**D** and **E**) Pathways and genes shown had a *P* < 0.05. (**F**) qPCR analysis of *Slc1a5* in Th17 cells. Data show mean ± SEM, *n* = 3–5 samples, ** *P* < 0.01, ANOVA with Tukey’s post hoc analysis. See Supplemental Figure 12.

Our data show that male sex and AR signaling decrease glutamine uptake and/or glutaminolysis in Th17 cells, yet it remains unclear how AR signaling decreases expression of glutamine transporters and enzymes. Earlier studies showed that trimethylation at histone 3 lysine 27 (H3K27me3) repressed genes necessary for Th17 function (40–42). Therefore, we explored if AR signaling modified H3K27me3 signatures in Th17 cells differentiated from WT male, WT female, and *Ar*^Tfm^ male mice using CUT&RUN (cleavage under targets and release using nuclease) sequencing. Principle component analysis indicated that our samples grouped together depending on sex and AR status (Supplemental Figure 12A). Overall, Th17 cells from males had decreased H3K27me3 marks compared with those from female and *Ar*^Tfm^ male mice (Figure 7A). Analysis of differentially methylated areas showed significant differences in Th17 cells from male and *Ar*^Tfm^ male mice with over 2,500 genes differentially methylated and in Th17 cells from male and female mice with over 3,000 genes differentially methylated (Figure 7, B and C). In comparing females to *Ar*^Tfm^ males, 1,326 genes were differentially methylated genes in Th17 cells (Supplemental Figure 12B). Using genomic regions enrichment of annotations tool (GREAT) (43, 44) to find differences in gene ontology (GO) pathways in Th17 cells from WT male versus *Ar*^Tfm^ male mice, we determined one of the most enriched pathways for H3K27Me3 in males was the “promotion of glutamine transport” (Figure 7D). This pathway was also one of the top hits in the GO analysis of Th17 cells from male versus female mice (Supplemental Figure 12C). These data indicate that AR signaling altered repressive marks around genes related to glutamine transport, including *Slc38a3* and *Slc1a5* (Figure 7E). Using quantitative PCR (qPCR), we confirmed that expression of *Slc1a5* was significantly decreased with in WT males compared with WT females and *Ar^Tfm^* males (Figure 7F). Together, these data indicate that AR signaling modifies H3K27me3 to decrease expression of genes important in glutamine uptake and glutaminolysis in Th17 cells.

### AR signaling reduces Th17 metabolism in human cells.

**Figure 8:**
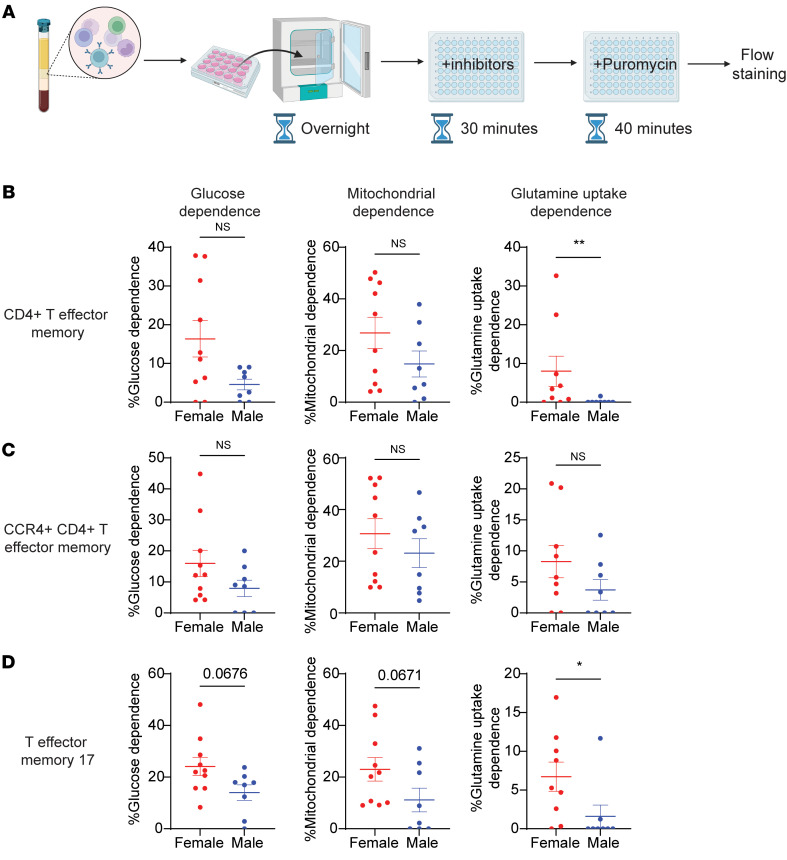
Males with severe asthma have decreased dependence on glutamine uptake in circulating CD4^+^ T cell subsets compared with females with severe asthma. (**A**) Model of SCENITH protocol on frozen PBMCs restimulated overnight with anti-CD3/CD28/CD2. (**B**–**D**) Glucose dependence, mitochondrial dependence, and glutamine uptake dependence in males and females measured using SCENITH inhibitors (2-deoxyglucose, oligomycin, and V9302, respectively) in (**B**) CD4^+^ TEMs (defined as Live, CD3^+^, CD4^+^, CD8^–^, CD45RA^–^, and CCR7^–^), (**C**) CCR4^+^ CD4^+^ TEMs, and (**D**) TEM 17 cells (defined as B and CCR4^+^, CCR6^+^). Data show mean ± SEM, *P < 0.05, **P < 0.01, or *P* value as shown; Mann-Whitney *U* test. See Supplemental Figure 13 and Supplemental Table 4.

Our murine in vitro and in vivo data demonstrated that AR signaling decreased expression of glutamine transporters and decreased glutaminolysis in Th17 cells, providing a mechanism for decreased reliance of glutaminolysis in male Th17 cells. We wanted to confirm this mechanism in circulating CD4^+^ T cells from men and women with severe asthma (Supplemental Table 4) using a single-cell metabolic approach, SCENITH (Figure 8A). (45) SCENITH is a flow-based single cell metabolic technique that relies on puromycin to track protein synthesis in the presence of metabolic pathway inhibitors — 2-deoxyglucose (2-DG) inhibiting glycolysis, oligomycin inhibiting mitochondrial respiration, and V9302 (46) inhibiting glutamine uptake to calculate the dependence of specific cells on these metabolic pathways (Supplemental Figure 13A). There were no significant differences between male and female CD4^+^ T cells in glucose dependence or mitochondrial dependence, but there was a significantly decreased dependence on glutamine uptake from male CD4^+^ T cells compared with female CD4^+^ T cells (Supplemental Figure 13B). Circulating CD4^+^ T effector memory cells (TEMs) had significantly decreased dependence on glutamine uptake via ASCT2 in male cells compared with female cells, but there were no sex differences in glucose or mitochondrial dependence (Figure 8B). Additionally, total CCR4^+^ cells had no significant differences in glucose, mitochondrial, or glutamine uptake dependence between male and female cells (Figure 8C). Circulating Th17 cells, defined as CCR4^+^ CCR6^+^ TEMs, from male individuals exhibited a trend toward decreased dependence on glucose and mitochondrial metabolism and a significant difference in glutamine uptake dependence for metabolism compared with Th17 cells from female individuals (Figure 8D). Other CD4^+^ T cells were examined, including naive CD4^+^ T cells. Naive CD4^+^ T cells from males had significantly decreased reliance upon glutamine uptake but no difference in glucose or mitochondrial dependences compared with naive CD4^+^ cells from female individuals (Supplemental Figure 13C). These data confirm that Th17 cells from males with asthma had decreased glutamine uptake compared with Th17 cells from females with asthma.

## Discussion

There is a female predominance in lupus, rheumatoid arthritis, and severe asthma (17, 18). CD4^+^ T cells are important drivers of inflammation in these diseases, yet, how sex hormones directly modify CD4^+^ T cell effector responses remains unclear. In this study, we employed in vitro and in vivo methods to show that AR signaling restricted allergen-induced airway inflammation and AHR as well as Th17 differentiation and effector function by decreasing glutamine uptake and glutaminolysis. Our findings identify potential precision medicine targets by elucidating differential mechanisms of T cell metabolism by sex hormone signaling.

Previous studies by our laboratory and others showed that AR signaling decreased Th17- and Th2-mediated inflammation (25, 26, 30, 47). Additionally, prior studies and our findings in this study show that AR signaling decreased Th17 cell differentiation and effector function (25, 26). In this study, we expanded these findings by showing a foundational mechanism: AR signaling decreased CD4^+^ T cell glutamine uptake and subsequent glutaminolysis, that male mice with normal androgen levels and AR signaling had decreased CD4^+^ T cell dependence on glutaminolysis pathways, and that CD4-specific deletion of *Gls* decreased Th2 and Th17-airway inflammation only in female mice. Further, we found that AR signaling decreased glutamine uptake transporters, such as ASCT2 (Slc1a5) or SNAT1 (Slc38a1). Glutamine uptake is a process that utilizes different transporters that are affected by a variety of environmental factors, providing scenarios that rely more heavily on Slc1a5 or Slc38a1. These results provide an intrinsic pathway for how AR signaling attenuates glutaminolysis leading to attenuated Th17 cell differentiation and effector function in males.

Glutaminolysis is important for Th17 development as well as Th17 and Th2 effector function (8, 12, 13). Importantly, we studied mediastinal lymph nodes collected from deceased donors and performed CyTOF (thus studying a specific tissue rather than peripheral blood) and discovered there was a sex bias in CD4^+^ T cell metabolic enzyme expression in various subsets. In these donors we also determined that GLUD1 expression was significantly decreased in Th2 and Th17 cells in the lymph nodes of men compared with women. Due to the nature of the samples, a direct effect of AR signaling could not be determined; however, future studies using patients undergoing gender affirming care (48) or patients with androgen insensitivity syndrome could provide this information. As our panel was limited by the available antibodies and panel size, we acknowledge that other pathways could potentially be regulated as well. Additionally, our analysis was limited to CD4^+^ T cells, but there are likely many differences in other immune cell types, which can be explored in future studies.

AR signaling decreasing glutamine uptake in CD4^+^ T cells, and subsequent glutaminolysis in Th17 cells results in AR signaling shunting glutamine metabolism away from the TCA cycle and ROS management through GSH. Increased ROS limited the Th17 differentiation and effector function by promoting a regulatory phenotype (6, 12, 49, 50). Our glutamine tracing studies indicate that differentiated WT male Th17 cells are less likely to have GSSG, reducing their ability to control ROS and thereby likely reducing Th17 differentiation and effector function. On the other hand, quantitative analysis on mitochondrial size, area, and roundness showed that Th17 cells from WT males had a slight but significant increase in area compared with Th17 cells from *Ar*^Tfm^ mice. These results are surprising and a mechanism for increased mitochondrial size but diminished mitochondrial flux (as measured by Seahorse) is not clear and warrants further studies.

Nutrient uptake is important in driving T cell differentiation and effector function (2, 3), but nutrient uptake is also important in other immune cell populations. ^18^F glutamine–uptake studies showed that AR-deficient mice had increased glutamine in CD4^+^ T cells as well as CD11b^–^ CD4^–^cells. It is possible that the CD11b^–^ CD4^–^ cells contain ILCs and/or γΔ T cells, providing additional cell subsets important in airway inflammation where AR signaling attenuates glutamine uptake. However, the magnetic separation technique used in this study prevents specific isolation of ILCs and γΔ T cell populations, so we cannot be certain what cell types are within the CD11b^–^ CD4^–^ population. Additional studies focusing on other cell types are warranted to determine how AR signaling, or other sex hormones, are modifying metabolism during ongoing inflammation.

While glutamine uptake and utilization became the focus of our study, we acknowledge that AR signaling could be driving differential uptake and utilization of other nutrients, as shown in the other significant hits in our metabolomics studies. Arginine and serine are important in Th17 effector programs and play a role in polyamine and carbon metabolism (10, 51). The differences in AR signaling in these pathways could further affect the Th17 differentiation and effector program and balance of T cells. This would provide exciting possibilities for future studies.

Increased methylation at H3K27me3 has been reported to decrease metabolic function in Th17 differentiation (41, 42). Further, previous studies showed the accessibility of these genes, especially regarding H3K27me3 via KDM6, is important in the differentiation and maintenance of Th17 effector function (42). This repressor function can impact a multitude of pathways, most important being mitochondrial biogenesis and respiratory activity. These findings, along with previous studies on the importance of mitochondrial dynamics and function in Th17 cells (52–54) align with our functional findings regarding decreased mitochondrial respiration and increased H3K27me3 marks around glutamine transport pathways. Therefore, it is possible that AR signaling impacts KDM6-mediated H3K27me3 methylation on multiple Th17-related pathways and this can be explored in future studies. Additional studies are also needed on the effect of AR signaling on acetylation pathways, as previous studies have indicated effects of polyamine metabolism on acetylation of regions related to CD4^+^ T helper differentiation (5). Further, we show several differentially methylated regions for cellular function and T cell activation based on AR signaling, indicating that methylation patterns regulated by AR signaling may be regulating Th17 differentiation and effector function through multiple pathways.

Many studies have indicated that both ovarian hormones and androgens directly affect the proliferation and function of macrophages, dendritic cells, T regulatory cells, CD8^+^ T cells, and CD4^+^ T cells (17, 18). Here, we show a dichotomized reliance upon a specific metabolic pathway based on AR signaling. We found that glutaminolysis is important in Th17 cell effector function in females individuals but may not have as large of an impact on Th17 cells from male individuals. This indicates that AR signaling, while decreasing glutaminolysis, may force Th17 cells to rely upon different metabolic pathways. Thus, exploring sex hormone signaling is essential in drug development, as more immunometabolism-targeted therapeutics are developed to target disease (55, 56). Our study indicates that those drugs may be differentially effective based on sex and the metabolic mechanisms that are driven by sex hormones. Our metabolomics data and CRISPR screens suggest glutaminolysis, and other pathways are differentially regulated by sex hormone signaling in Th17 cells. Future studies on immunometabolism and the targeting of immunometabolism for therapeutics should determine the sex-specific effects of the drugs and how that may impact efficacy. Additionally, the focus of this study on androgens supports the use of weak androgens, such as inhaled dehydroepiandrosterone (DHEA) or sulfonated DHEA (DHEA-S), as possible therapeutics or adjuvant therapy to increase the effectiveness of other therapeutics (57–59). Further studies on the effect of sex hormones on specific metabolic pathways can lead to new targets for therapy and increased effectiveness for those therapeutics, which would further the field of precision medicine.

## Methods

### Sex as a biological variable.

Our study examined immune cells from human males and females as well as male and female mice. Sexually dimorphic effects are reported in the text.

### Human samples.

Single-cell suspensions of deidentified human lung draining lymph nodes were used with no information on use of exogenous sex hormones, including birth control medications, pregnancy, breastfeeding, and/or phase of menstrual cycle provided. Patient demographic information and cause of death are provided in Supplemental Table 2.

Human PBMCs were collected from patients with severe asthma as defined by the American Thoracic Society criteria (60). Patients were 18–45 years old and were excluded if they had any symptoms of infection within the past week, comorbid Th17-associated disease (e.g., Crohn’s disease), or administration of systemic exogenous hormones (e.g., oral contraceptives, estrogen replacement therapy). Women were excluded if they were pregnant, breastfeeding, menopausal, or had undergone an oophorectomy or hysterectomy. Patient demographic information is provided in Supplemental Table 4 and as stated in the text. PBMCs were isolated from whole blood using SepMate-50 PBMC Isolation Tubes (STEMCELL Technologies) and Lymphoprep (STEMCELL Technologies) density gradient isolation. A red blood cell lysis (BioLegend) was performed according to manufacturer instructions. Cells were aliquoted at 10 million cells/mL in BAMBANKER serum-free cell freezing medium (Bulldog Bio) and preserved in liquid nitrogen.

### Mice.

Mice were housed in a pathogen-free facility with a maximum of 5 mice per cage and ad libitum access to food and water. C57BL/6J WT (no. 000664), *Ar^Tfm^* (no. 001809), *Ar^fl/f^* (no. 018450)*^l^*, *Esr1^fl/fl^* (no. 032173), and *Rag1^–/–^* (no. 002216) mice were obtained from Jackson Laboratories and bred in house. OT-II Cas9 double transgenic mice, *Gls^fl/fl^* and *Cd4^Cre^ Gls^fl/fl^* were provided in-house. *Cd4^Cre^* mice that were crossed to *Ar^fl/fl^* mice were provided by the lab of Holly Algood (Vanderbilt University, Nashville, Tennessee, USA). Mice were genotyped, and male and female naive mice were used as described below. For GNX experiments, mice were gonadectomized at 3–4 weeks of age, around the time of puberty in mice. In select experiments, flutamide and vehicle pellets (50 mg, 60-day release) were obtained from Innovative Research of America and inserted into 8–10 week old mice for 3 weeks prior to starting HDM challenges. For all other experiments, mice were 8–12 weeks old mice at the start of the experiment.

### Cell lines.

Plat-E retroviral packaging cell line (ATCC) was maintained at 37°C with 5% CO_2_ in DMEM media (Gibco) supplemented with 10% FBS (R&D Systems), 100 U/mL penicillin/streptomycin (Gibco), 1 μg/mL puromycin (Gibco), and 10 μg/mL blasticidin (Gibco) to maintain expression of viral packaging genes.

### CyTOF on human lymph nodes.

CYTOF was run by the Vanderbilt University Cancer and Immunology Core (CIC) on human lung draining lymph node cells using the panel located in Supplemental Table 1 (with antibody manufacturer in Supplemental Table 5) and analysis pipeline previously described (8, 61, 62). T cell subsets were identified using surface markers and metabolic enzyme expression was quantified using mean expression analysis. Median expression for metabolites was determined by the arcsinh transformed ratio of median versus the lowest sample in the analysis.

### In vivo HDM challenge protocol.

Mice were challenged intranasally with 40 μg of HDM (Greer) 3 times per week for 3 weeks. Twenty-four hours after the last challenge, mice were sacrificed for endpoint analysis.

### BAL collection.

BAL was collected using 800 μL of PBS and staining cells adhered to a slide using the 3-Step Stain kit (Richard-Allen Science, Thermo Fisher Scientific). (25) Eosinophils, neutrophils, macrophages/monocytes, and lymphocytes were counted as previously described. (25)

### ELISAs.

Homogenized left lungs or BAL supernatants were used to measure IL-13 and IL-17A levels with Quantikine kits (R&D) per manufacturer’s instructions. Any value below the limit of detection was assigned half the value of the lowest detectable standard.

### Flow cytometry.

Lungs were harvested, minced, and digested as previously described (8, 26). In select experiments, cells were restimulated with 1 μM ionomycin (Sigma-Aldrich), 50 ng/mL PMA (Sigma-Aldrich), and 0.07% Golgi Stop (BD Biosciences) at 37°C for 4 hours, then stained for flow cytometry analysis as previously described using antibodies in Supplemental Table 5 (8, 26). For detection of mitochondrial superoxide or MitoTracker Green, Th2 and Th17 cells were washed and stained with surface markers and MitoSox Red (2.5 μM, Thermo Fisher Scientific) or MitoTracker Green (2.5 μM, Thermo Fisher Scientific). Flow cytometry was conducted on a Cytek Aurora (Cytek Biosciences) and data were analyzed using FlowJo (BD Biosciences) with mean fluorescence intensity (MFI) calculated using the geometric mean.

### Airway hyperresponsiveness.

Mice were anesthetized with pentobarbital sodium (85 mg/kg) and AHR to methacholine (0–100 mg/mL; Sigma-Aldrich) was conducted using the Sci-Req FlexiVent machine, as previously described (26).

### In vitro CD4^+^ T cell activation and differentiation.

Splenic murine naive CD4*^+^* T cells were isolated using the STEMCELL Mouse Naive CD4*^+^* T Isolation Kit (STEMCELL Technologies) instructions. Naive T cells were plated at 500,000 cells per well in a 24-well nontissue-culture plate coated with anti-CD3 (1 μg/mL, BD Biosciences cat. 553057) and anti-CD28 (0.5 μg/mL, BD Biosciences cat. 553295). Cells were cultured at 37°C with 5% CO_2_ in IMDM with GlutaMax and HEPES (Gibco), 10% FBS (R&D Systems), 1% penicillin/streptomycin (Gibco), 1% sodium pyruvate (Gibco), 50 μM 2-mercaptoethanol (Sigma-Aldrich), and appropriate differentiation cytokines and antibodies for 3 to 4 days. For Th17 differentiation, differentiation media included rhTGF-β (0.5 ng/mL; Peprotech cat. 100-21), rmIL-23 (10 ng/mL; R&D Systems cat. 1887-ML-010/CF), rmIL-6 (40 ng/mL; Peprotech 216.16), anti-IL4 (10 μg/mL; BioLegend cat. 504122), and anti-IFNγ (10 μg/mL; BioLegend cat. 505847). For Th2 differentiation, differentiation media included rmIL-4 (10 ng/mL; Peprotech cat. 214-14) and anti-IFNγ (10 μg/mL; BioLegend cat. 505847).

### Seahorse extracellular flux assays.

Seahorse extracellular flux assays were performed on the Seahorse XFe96 Analyzer (Agilent) using the Seahorse XFe96 Extracellular Flux Assay Kits (Agilent), as previously described (8, 33). For the Substrate Oxidation Assay, CB-839 (MedChemExpress, 10 μM) was used to determine the acute effects of GLS inhibition on mitochondrial respiration. All assays were normalized using cell counts acquired via bright field imaging using a Cytation 5 Imager (BioTek; Supplemental Figure 7).

### Mass spectrometry targeted metabolomics.

Th17 cells were lysed and polar metabolites were extracted with 500 μL methanol with the internal standards (13C2-Tyr and 13C3-Lactate, Cambridge Isotope Lab). Supernatants were evaporated to dryness under a gentle stream of nitrogen gas and reconstituted in 100 μL of acetonitrile/water (2:1). Metabolites were then measured by a targeted HILIC-MS/MS method developed at the Vanderbilt Mass Spectrometry Research Center (108 individual metabolites). Individual reference standards of all analytes were infused into the mass spectrometer for the optimization of ESI and selected reaction monitoring (SRM) parameters. LC-MS analysis was performed using an Acquity UPLC system (Waters) interfaced with a TSQ Vantage quadrupole mass spectrometer (Thermo Fisher Scientific). Pathway analysis and statistical significance was measured using MetaboAnalyst 5.0 (37).

### Transmission electron microscopy.

Th17 cells were differentiated for 3 days, washed with warm PBS 2 times, fixed in 2.5**%** glutaraldehyde in 0.1 M cacodylate for 1 hour at room temperature, and stored at 4°C for 24 hours. Cells were processed and prepared for transmission electron microscopy (TEM) as previously described (63). TEM was performed using a Tecnai T12 operating at 100 kV with an AMT NanoSprint CMOS camera using AMT imaging software for single images. Quantification for mitochondrial area, roundness, and density was conducted on Fiji by manually segmenting all mitochondria within cell cross sections from tiled TEM datasets on 100–110 mitochondria per sample.

### CRISPR Screening.

CRISPR screen library design and preparation were completed as previously described (10). The glutamine library contained 32 total gene targets with 138 gRNAs. Using Gibson assembly master mix, oligos were cloned with the pMx-U6-gRNA-BFP vector. Plat-E retroviral packaging cells were transfected with gRNAs to package DNA into retrovirus. Naive T cells were isolated from male and female OTII-Cas9 mice and differentiated as above except for T cell activation occurring through presentation of OVA peptides 323–339 (Sigma-Aldrich) by irradiated splenocytes from WT mice. 2 days after activation, T cells were retrovirally transduced with retronectin-coated plates (Takara Bioscience). Transduced OTII-Cas9 Th17 cells were adoptively transferred into *Rag1*^–/–^ female mice that were challenged every other day with 25 μg of OVA protein on day –1 (prior to adoptive transfer) through day 7. Twenty-four hours after the last challenge, lungs were harvested, digested, and processed for gRNA frequencies as above and as previously described (10). All samples maintained at least 1,000-fold representation of the library through all processing. FASTQ files were analyzed using the Model-based Analysis of Genome-wide CRISPR/Cas9 Knockout (MAGeCK v0.5.0.3) method to determine statistically significant gRNA enrichments or depletions (64).

### ^13^C Targeted Metabolomics.

Th17 cells were plated in glutamine free RPMI-1640 containing 4.05 mM ^13^C_5_-glutamine (Cambridge Isotope Laboratories) for 4 hours. After treatment with heavy labeled glutamine, cells were washed with PBS, pelleted, flash frozen in liquid nitrogen, and stored at –80°. Metabolites were extracted, spiked with 100 nmol ^13^C-1-Lactate (internal standard), and precipitated protein was dried under nitrogen as previously described (65). Chromatography was performed and scheduled MRM using an AB SCIEX 6500 QTRAP with the analyte parameters shown in Supplemental Table 6 and as previously described. All analytes were quantified via LC-MS/MS using the ^13^C-1-Lactate internal standard and normalized to the protein in each respective sample’s cell pellet. Outliers were removed using interquartile range.

### ^18^F-Glutamine PET-CT imaging and glutamine uptake assay.

WT female, WT male, and/or *Ar^Tfm^* male mice were challenged with HDM. PET imaging studies and glutamine uptake in the cell types were performed as previously described using 1 mCi of ^18^F-glutamine synthesized at Vanderbilt University Medical Center (39, 66). Twenty-four hours after their last challenge, the mice were injected with 1mCi of ^18^F-glutamine and rested for 40 minutes before lungs and spleens were collected, digested, and processed to a single-cell suspension as described above. Cell suspensions were fractionated using magnetic CD11b-positive selection for depletion followed by CD4-positive selection (Miltenyi Biotec) (39). Fractions were resuspended in 1 mL medium and used for viable cell counts using trypan blue, flow cytometry for fraction composition, and radioactivity measurement using the Hidex Automatic γ Counter. To determine per cell ^18^F-glutamine activity, time-normalized counts per minute (CPM) measured on the γ counter were divided by the number of viable cells.

### CUT&RUN.

CUT&RUN was performed on Th17 cells using the CUT&RUN kit with magnetic bead–based method per manufacturer instructions (Cell Signaling Technology). Briefly, 200,000 Th17 cells were washed and resuspended in wash buffer and mixed with 10 μL preactivated ConA beads. The cell-bound beads were rotated with 2.5 μL of H3K27me3 antibody or 5 μL of IgG control for 2 hours at 4°C. Beads were then washed with 1 mL digitonin buffer, resuspended in 100 μL digitonin buffer mixed with 1.5 μL pA-MNase and rotated for 1 hour at 4°C. Cells were washed 2 times with 1 mL digitonin buffer, resuspended in 150 μL digitonin buffer containing 2 mM CaCl_2_, and incubated for 30 minutes in an ice/water mix. 150 μL of stop buffer was added and beads were incubated at 37°C for 10 minutes to free fragments from beads. Using the KAPA HyperPrep Kit (Roche), DNA was extracted and constructed into sequencing libraries. Libraries were sequenced for 150 cycles in paired-end mode on the Illumina Nova-seq 6000 platform by VANTAGE. Analysis was performed as previously described (10). Briefly, fragments were trimmed using Trimmomatic (v0.39) and mapped onto mouse genome GRCm38.p6 using bowtie2 (v2.3.5.1). Alignment SAM files were transformed into BAM and bigwig files using samtools (v.9) and deeptolls (v3.3.1). MACS2 (v2.2.7.1) was used for calling peaks and DiffBind compared differential peaks between groups to make the MA plots. GREAT (v4.0.4) was used to determine significantly different GO pathways and genomic regions based on region-based binomial q value FDR < 0.05.

### Real-time PCR assay.

Total RNA was extracted using the RNeasy Mini Kit (Qiagen). cDNA was prepared using the SuperScript IV First-Strand Synthesis System (Thermo Fisher Scientific) and normalized to 400 ng of total RNA. Gene expression was determined using TaqMan primers and the TaqMan Universal Master Mix purchased from Applied Biosystems, and relative expression was normalized to *Tbp* (encoding TATA-box binding protein).

### SCENITH.

PBMCs were plated at 1 million cells per well in a 24-well nontissue culture–treated plate as described above in the CyTOF section. T cells were activated overnight at 37°C with 5% CO_2_ using Human T Cell Activation/Expansion Kit (Miltenyi) containing antibodies to CD3, CD28, and CD2 with a 1:20 bead to cell ratio. Cells were then plated in 96-well nontissue culture–treated U bottom plates at 400,000 cells per well in 100 μL media and allowed to rest for 1 hour at 37°C with 5% CO_2_. Cells were then treated with appropriate inhibitors or vehicle for 30 minutes (DMSO, Sigma-Aldrich) as follows: 2-deoxyglucose (100 mM, Cayman Chemical), oligomycin (1.5 μM, Cayman Chemical), and/or V9302 (10 μM, H. Charles Manning) (46). After 30 minutes, cells were treated with puromycin (10 μM, Santa Cruz Biotechnology) for 40 minutes. Cells were then washed with PBS and stained with Live Dead Aqua fixable viability dye for 20 minutes at 4°C. Subsequently, cells were washed with PBS with 3% FBS and stained for surface markers at 4°C as indicated. Cells were washed again with PBS with 3% FBS and fixed using the Foxp3 Transcription Factor Fix/Perm Kit (Thermo Fisher Scientific) per manufacturer instructions. Cells were washed with Perm Buffer and stained with an anti-puromycin antibody (Sigma-Aldrich cat. MABE343-AF488) at 1:1,000 at 4°C. Finally, cells were washed with Perm Buffer and PBS with 3% FBS and flow cytometry was run using a MACSQuant 16 (Miltenyi Biotec). Analysis was done using FlowJo and as previously described (45).

### Statistics.

Statistical analyses were performed using GraphPad Prism (v. 9 or 10) unless noted. Data are represented as mean ± SEM. Data were significant when *P* was less than 0.05. Outliers were identified using the GraphPad Prism 9 ROUT method with a Q of 0.5% (α of 0.005). Comparisons between 2 groups were performed using a 2-way *t* test or Mann-Whitney *U* test for parametric or nonparametric data, respectively. Comparisons of greater-than 2 groups were made using an ANOVA with Tukey’s post hoc for multiple comparisons. For targeted metabolomics, pathway analysis was conducted using MetaboAnalyst 5.0, GlobalTest for pathway enrichment analysis, and adjusted for multiple testing and the FDR and pathway impact calculated using pathway topology analysis. For CRISPR analysis, we used MAGeCK (v0.5.0.3) as previously described (64). For CUT&RUN, MACS2 (v2.2.7.1) was used for calling peaks and DiffBind compared differential peaks between groups to make the MA plots. GREAT (v4.0.4) was used to determine significantly different GO pathways and genomic regions based on region-based binomial q value FDR < 0.05.

### Study approval.

All studies were conducted in accordance with the Helsinki principles under protocols approved by the VUMC IRB (protocols 122554 and 202162). All mouse procedures were performed under VUMC IACUC–approved protocols and conformed to all relevant regulatory standards.

### Data availability.

All data are available in the Supporting Data Values file. CUT&RUN sequencing data have been deposited in the Gene Expression Omnibus (GEO) database (GSE241823).

## Author contributions

NUC, DCN, and JCR designed the research. NUC, JYC, EHP, MZM, MMW, SNK, KEM, EQJ, ENA, DRH, CC, AS, MTS, KV, XY, KS, ESK, and VDG performed the research and provided essential technical support. JCR, RSP, KNC, RDG, and AIS provided essential expertise, materials, and samples. NUC, JYC, EHP, MMW, MZM, SNK, KEM, EQJ, ENA, CC, KS, ESK, XY, and DCN analyzed data. NUC, JYC, and DCN wrote the paper with contributions from the other authors.

## Supplementary Material

Supplemental data

Supporting data values

## Version 1 10/15/2024

In-Press Preview

## Version 2 12/02/2024

Electronic publication
