# Supplementary material for: Androgen signaling restricts glutaminolysis to drive sex-specific Th17 metabolism in allergic airway inflammation
Source: J Clin Invest. 2024 Oct 15;134(23):e177242. doi: 10.1172/JCI177242 (PMC11601904; doi:10.1172/JCI177242)
Supplement: Supplemental data [file jci-134-177242-s101.pdf]

Supplemental Information for Androgen Signaling Restricts Glutaminolysis to Drive Sex-Specific Th17 Metabolism in Allergic Airway Inflammation

Nowrin U Chowdhury[1,2], Jacqueline-Yvonne Cephus[3], Emely Henriquez Pilier[1,2], Melissa M Wolf[1,2], Matthew Z Madden[1,2], Shelby N Kuehnle[3], Kaitlin E McKernan[1,2], Erin Q. Jennings[2,3], Emily N Arner[2,3], Darren R. Heintzman[1,2], Channing Chi[1], Ayaka Sugiura[1,2], Matthew T Stier[2,3], Kelsey Voss[1,2], Xiang Ye[1], Kennedi Scales[3], Evan S Krystofiak[4], Vivek D Gandhi[3], Robert D. Guzy[5], Katherine N Cahill[3], Anne I Sperling[6], R. Stokes Peebles, Jr.[3], Jeffrey C Rathmell[1,2], and Dawn C Newcomb[1,2,3]

[1]Department of Pathology, Microbiology, and Immunology, Vanderbilt University Medical Center, Nashville, TN, USA

[2]Vanderbilt Center for Immunobiology, Vanderbilt University Medical Center, Nashville, TN, USA

[3]Department of Medicine, Vanderbilt University Medical Center, Nashville, TN, USA

[4]Department of Cellular and Molecular Biology, Vanderbilt University, Nashville, TN, USA

[5]Department of Medicine, University of Wisconsin, Madison, WI, USA

[6]Department of Medicine, University of Virginia, Charlottesville, VA, USA

Corresponding Author:

Dawn C. Newcomb, PhD

T-2220 Medical Center North

1161 21st Avenue South

Nashville, TN 37232

Dawn’s office number (615) 875-7782

dawn.newcomb@vumc.org

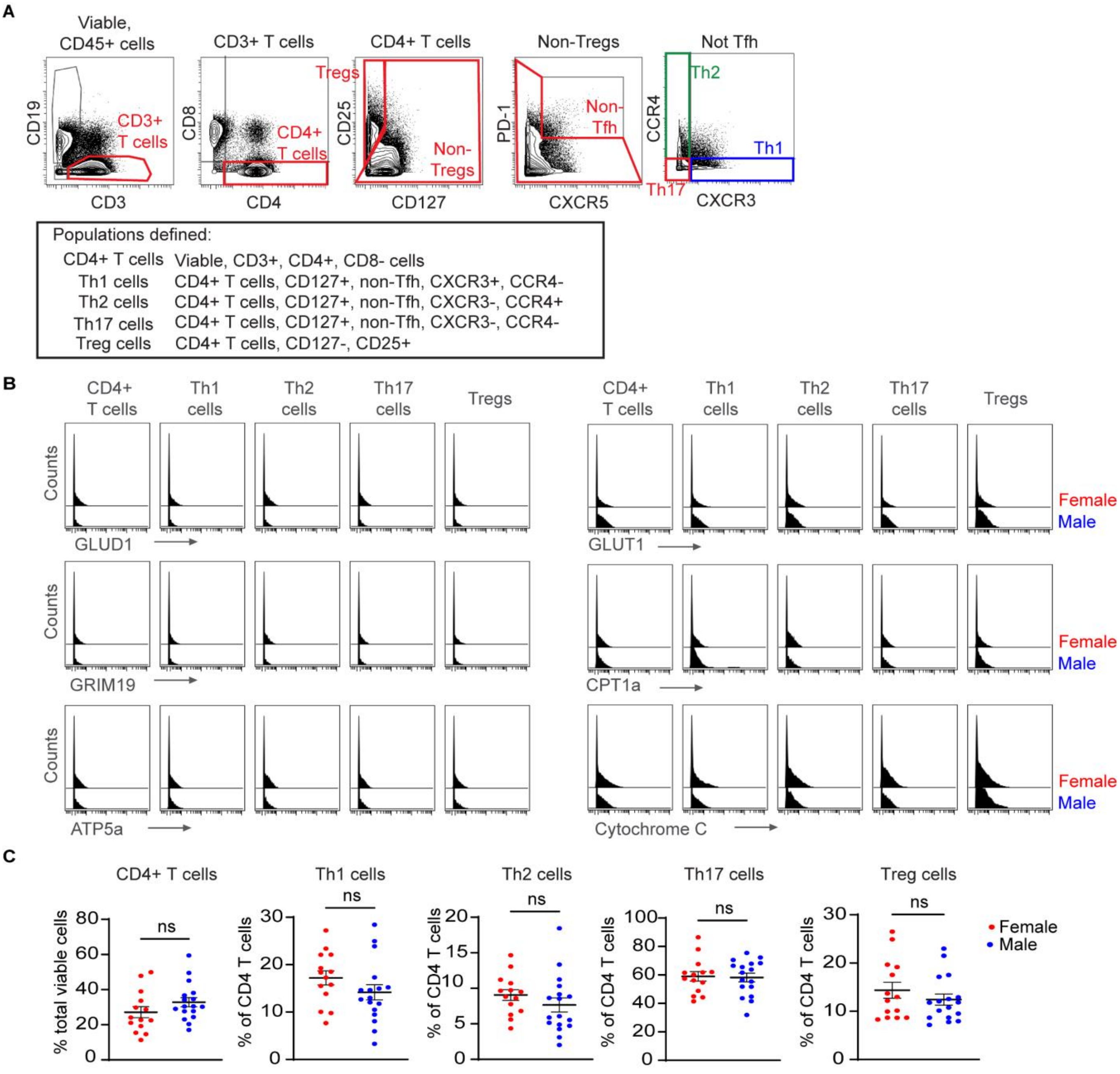

Supplemental Figure 1. Sex differences in metabolic marker expression in lung draining lymph nodes. Related to Figure 1. CYTOF was conducted on human lung draining lymph nodes from female (n=14) and male (n=17) deceased donors. **(A-B)** Gating strategy for CYTOF and CD4+ T cell populations defined. **(C)** Quantification of percentage of cell subsets in lung draining lymph nodes. n=14-17, mean ± SEM. $^*p<0.05$, ns: not significant, two-tailed Mann-Whitney U test.

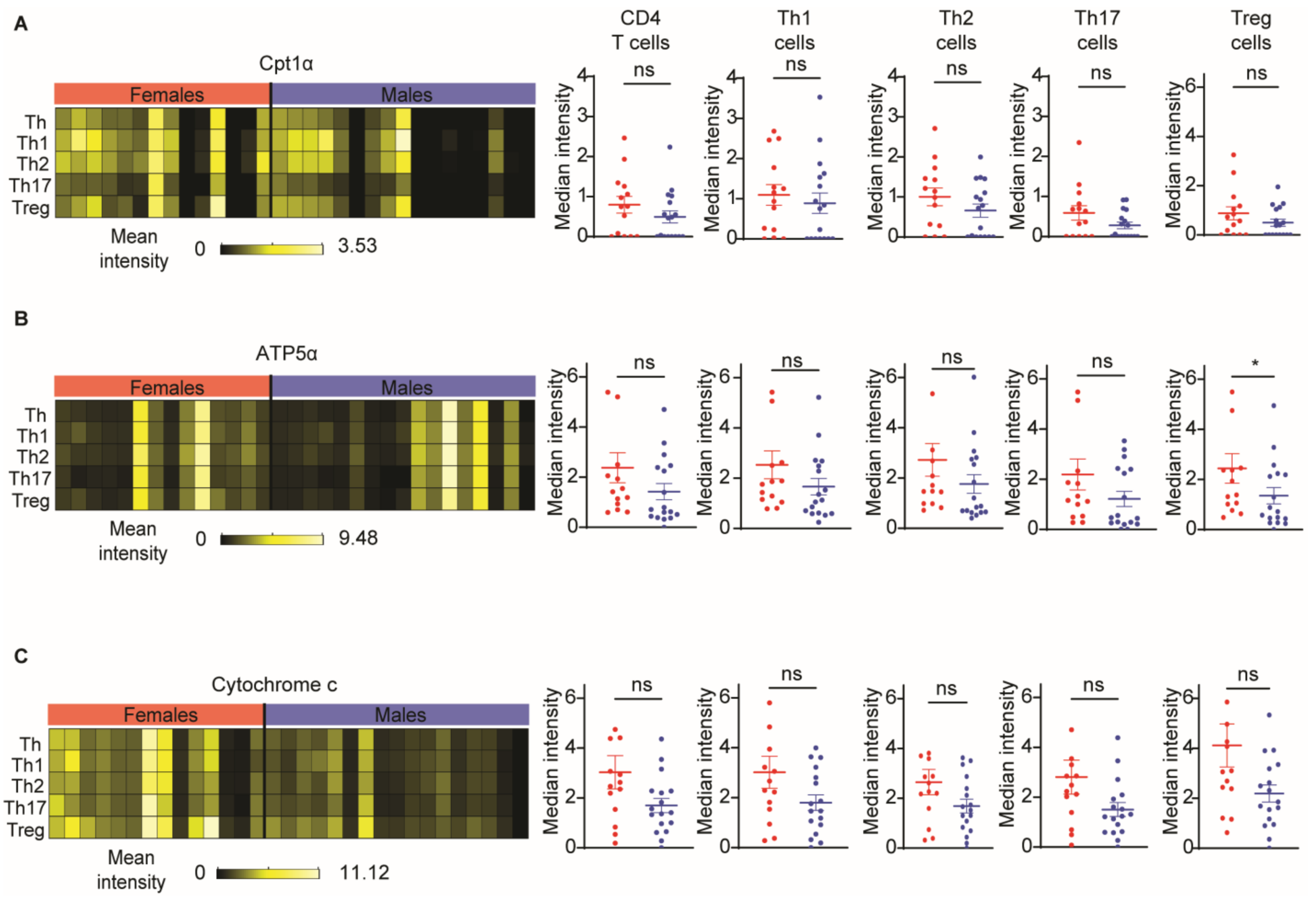

Supplemental Figure 2. CYTOF of lung draining lymph nodes reveals sex difference in ATP5a expression in Tregs. Related to Figure 1. CyTOF was conducted on human lung draining lymph nodes from de-identified female (n=14) and male (n=17) deceased donors. **(A-C)** Median expression of metabolic markers on CD4+ T cells, Th1, Th2, Th17, and Treg cells. Gating strategies and population definitions are shown in Figure S1. (mean ± SEM). *p<0.05, ns: not significant, two-tailed Mann-Whitney U test. See Supplemental Figure 1 and Supplemental Tables 1-2.

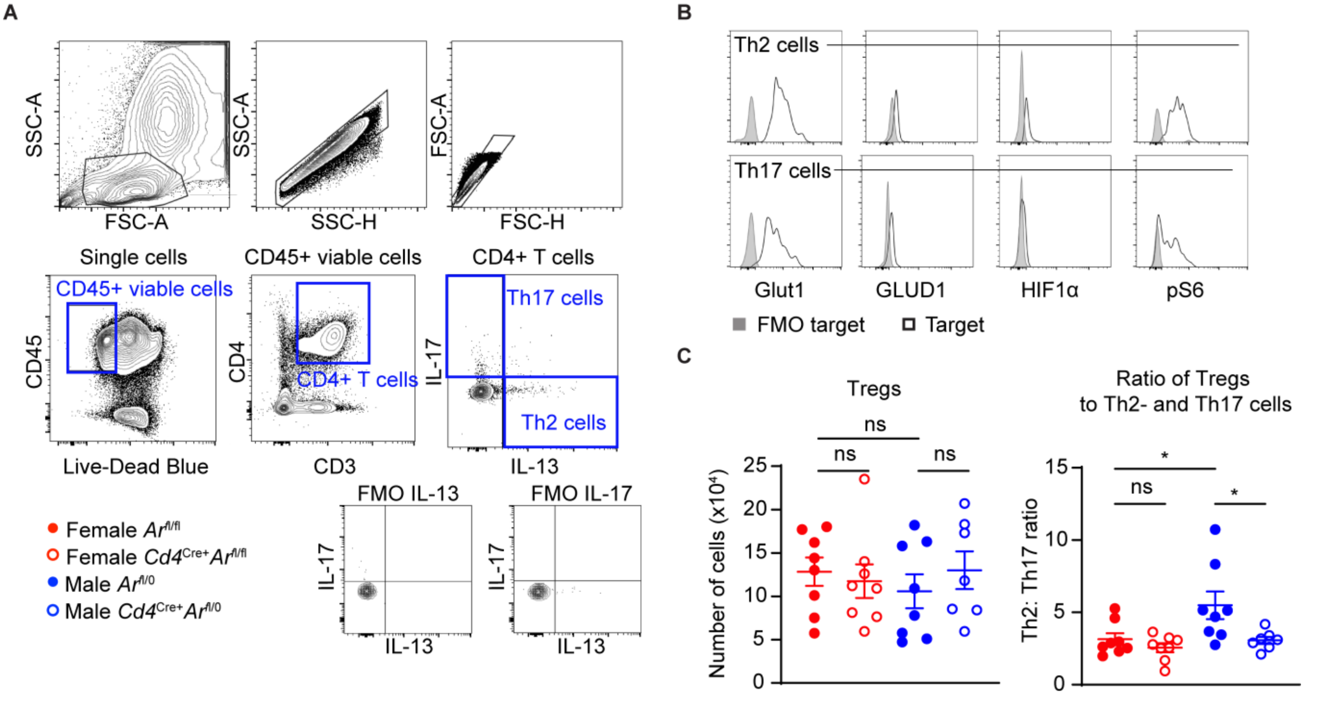

Supplemental Figure 3. Gating strategy for identification of Th2, Th17, and Tregs in HDM- challenged mice. Related to Figure 2. **(A-B)** Gating strategies for lung Th2 (IL-13+ CD4+ T cells) and Th17 cells (IL-17A+ CD4+ T cells) and expression of metabolites. **(C)** Quantification of Treg cell number and ratio of Tregs to Th2- and Th17 cells. Data was expressed as mean ± SEM: n=7-9 mice per group combined from 2 independent experiments. *p<0.05, ns: not significant, ANOVA with post-hoc Tukey testing.

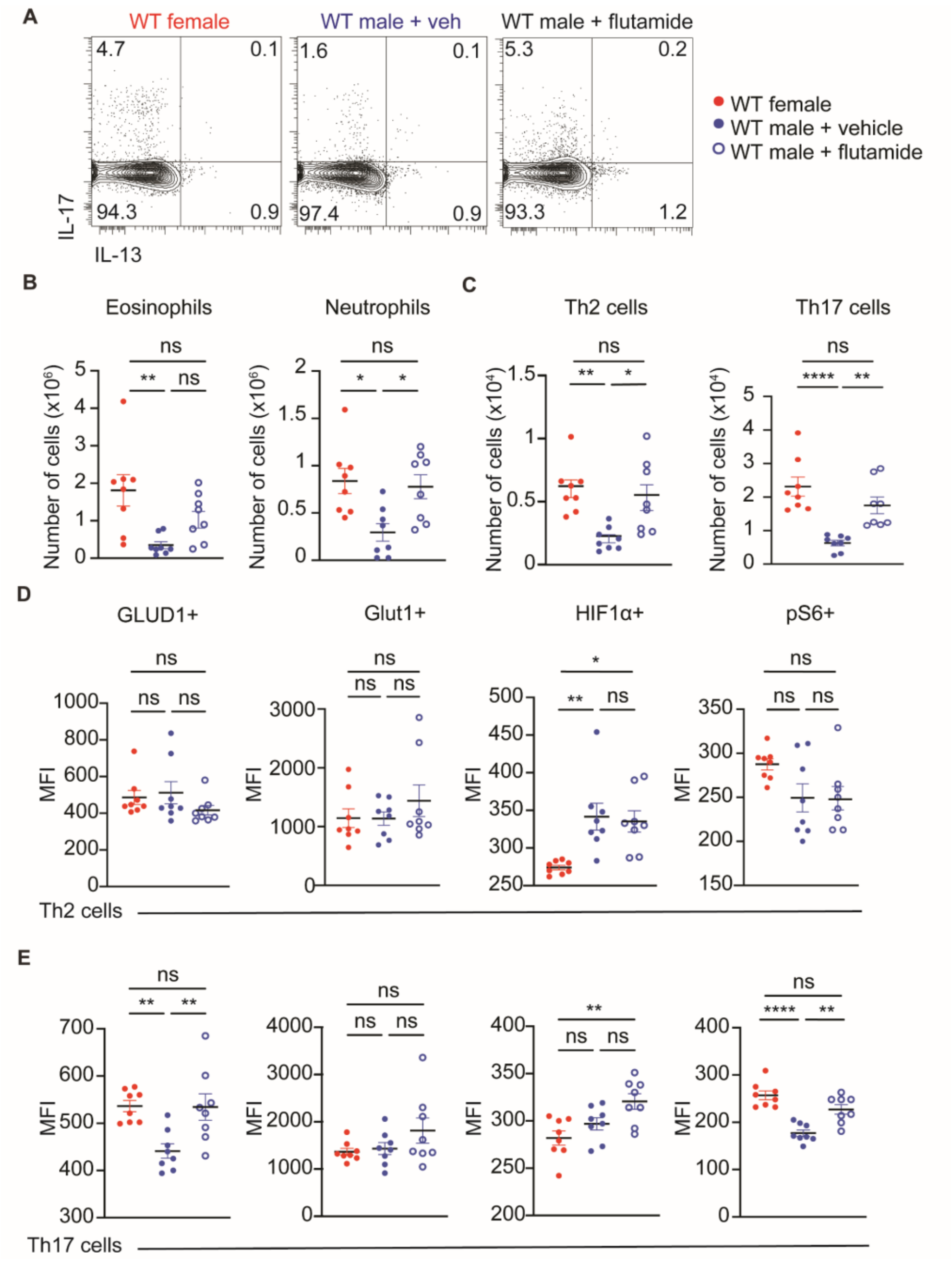

Supplemental Figure 4. Flutamide, an AR antagonist, increased HDM-induced Th17-mediated airway inflammation. Related to Figure 2. WT males were subcutaneously implanted with a vehicle or 50mg flutamide pellet for 3 weeks. HDM induced airway inflammation was induced on WT female, WT male + vehicle pellet, and WT male + flutamide pellet as described in Figure 2A. **(A)** Representative flow plots of IL-13+ Th2 cells and IL-17A+ Th17 cells. **(B)** Quantification of eosinophils and neutrophils in the BAL fluid from mice. **(C)** Quantification of lung Th2 and Th17 cells after HDM challenge. **(D-E)** Expression of GLUD1, Glut1, HIF1α, and pS6 in lung Th2 cells (top) and Th17 cells (bottom) after HDM challenge. Data are expressed as mean ± SEM: n=7-8 mice per group. *p<0.05, **p<0.01, ****p<0.0001, ns: not significant, ANOVA with post-hoc Tukey testing.

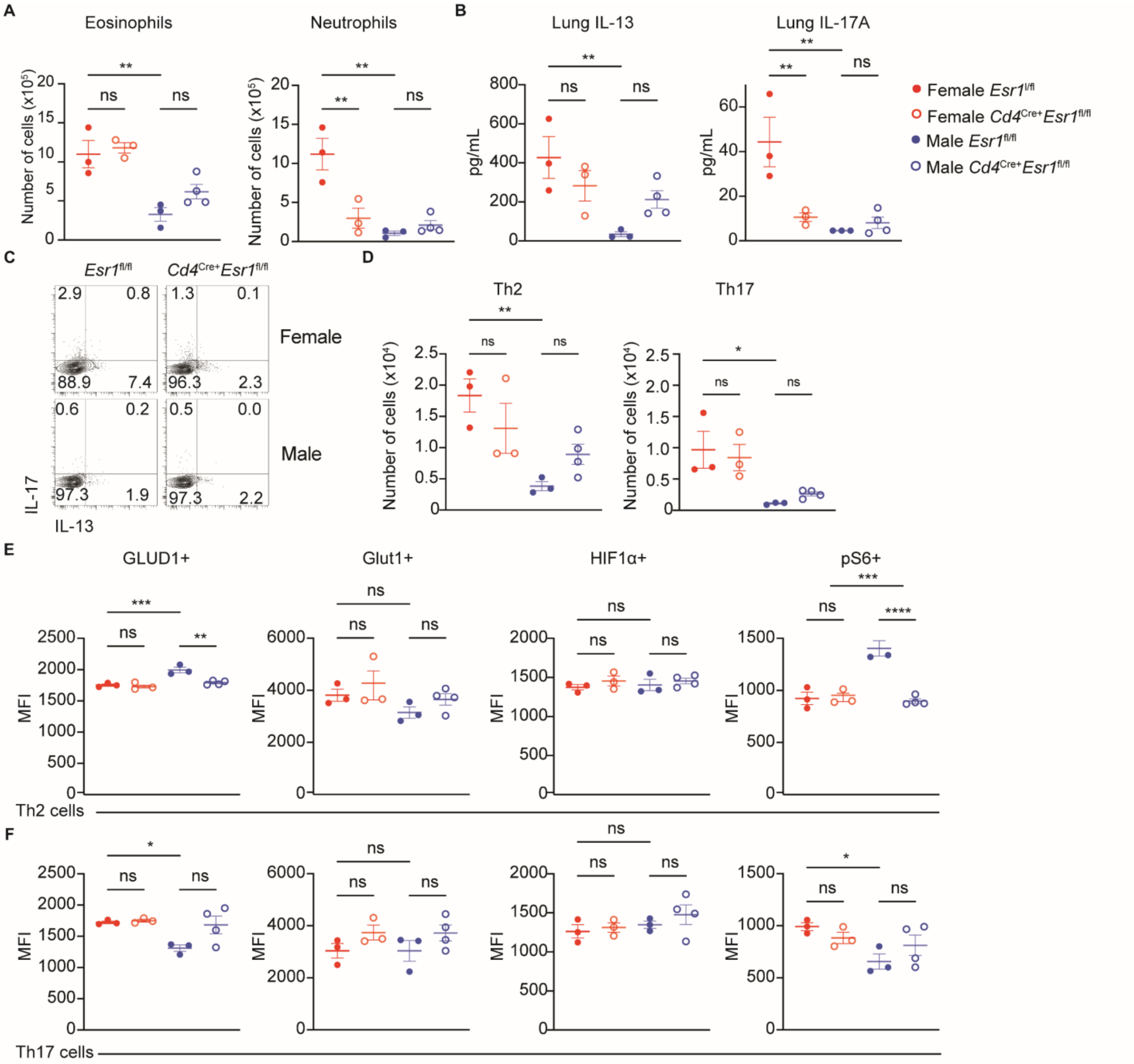

Supplemental Figure 5. Estrogen receptor alpha (ERα) signaling in CD4+ T cells increases neutrophil infiltration and Th17 cells in the lung, but does not impact the expression of metabolic enzymes in Th17 cells. Related to Figure 2. HDM induced airway inflammation was induced on female and male $Esr1^{fl/fl}$ *and* $Cd4^{Cre+}$ $Esr1^{fl/fl}$ mice as described in Figure 2A. **(A)** Quantification of eosinophils and neutrophils in the BAL fluid from mice. **(B)** IL-13 and IL-17A protein expression in whole lung homogenate. **(C)** Representative flow diagrams of IL-13+ Th2 cells and IL-17A+ Th17 cells. **(D)** Quantification of Th2 and Th17 cells in the lung. **(E-F)** Expression of GLUD1, Glut1, HIF1α, and pS6 in lung Th2 cells (top) and Th17 cells (bottom) after HDM challenge. Data are expressed as mean ± SEM: n=3-4 mice per group. *p<0.05, **p<0.01, ***p<0.001, ****p<0.0001, ns: not significant, ANOVA with post-hoc Tukey testing.

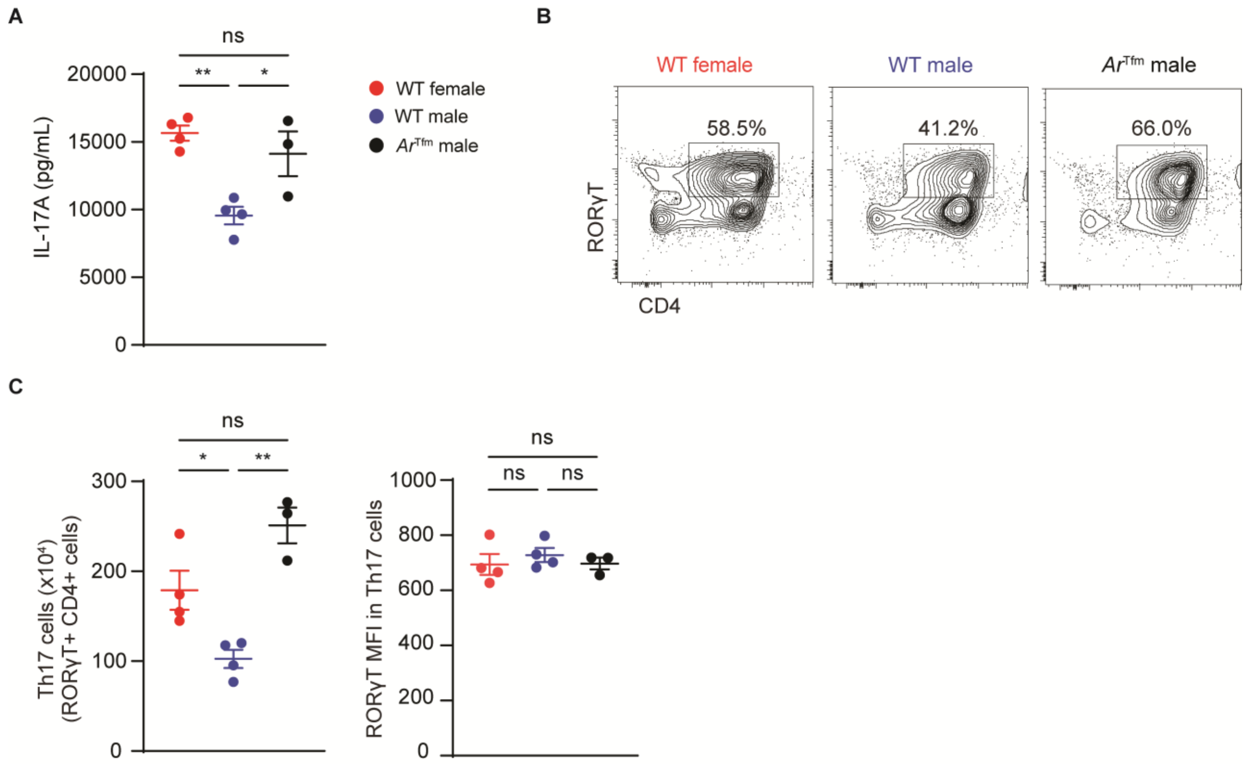

Supplemental Figure 6. AR signaling decrease IL-17A production and Th17 cell differentiation. Related to Figure 3. Naïve CD4+ T cells were isolated from spleens of WT female, WT male, and Ar^{Tfm} male mice and differentiated into Th17 cells. Cells and culture supernatants were collected on day 4. **(A)** IL-17A production in culture supernatants. **(B)** Representative flow plots of RORγT+ CD4+ T cells (Th17 cells). **(C)** Total numbers of RORγT+ CD4+ T cells (Th17 cells) were quantified by flow cytometry as well as the MFI for RORγT in Th17 cells. Data are expressed as mean ± SEM: n=3-4 mice per group. *p<0.05, **p<0.01, ns: not significant, ANOVA with post-hoc Tukey testing.

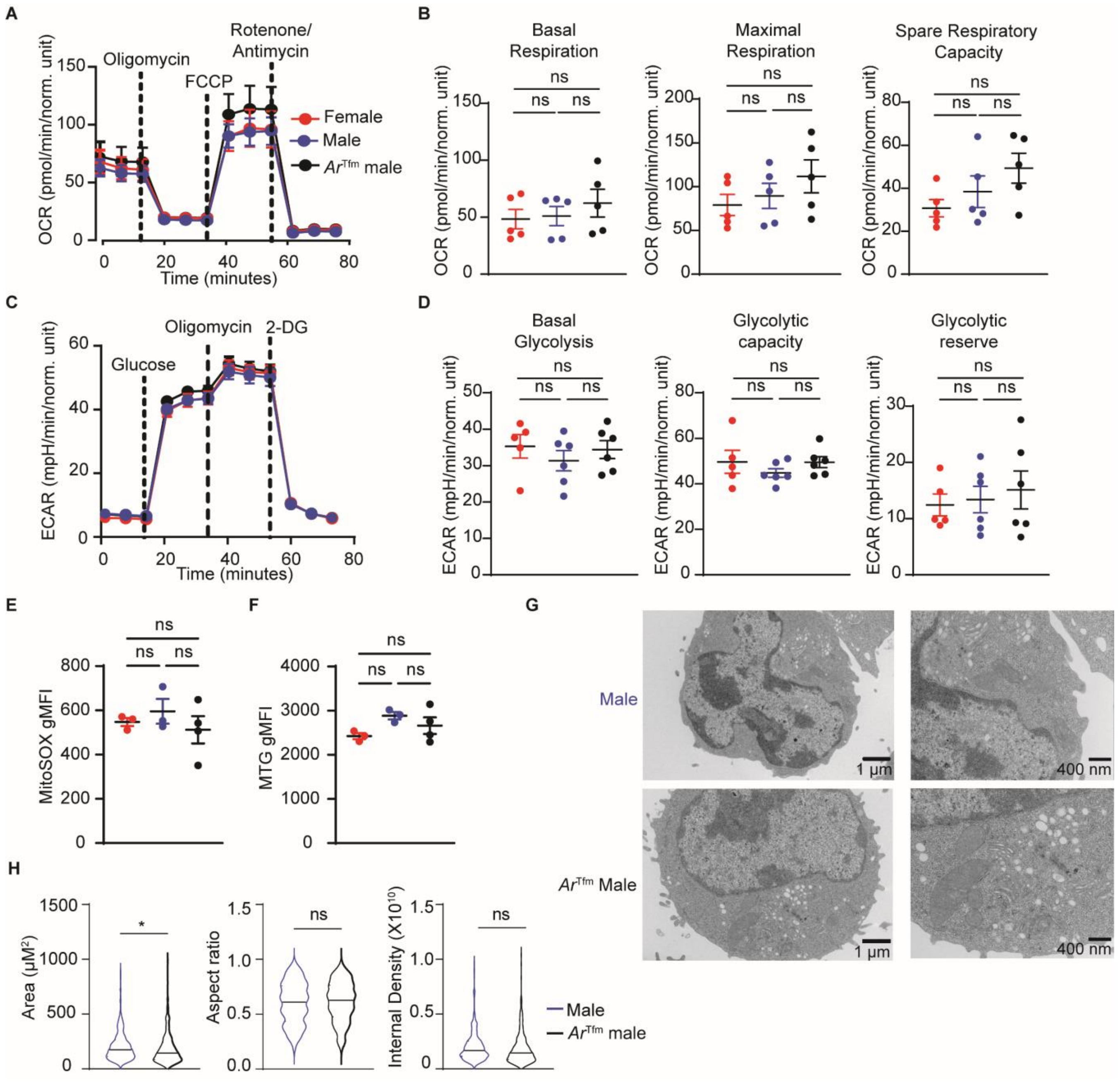

Supplemental Figure 7. AR signaling does not impact Th2 metabolism and EM quantification of Th17 cells. Related to Figure 3. (A) Seahorse MitoStress Test on differentiated Th2 cells from WT female, WT male, and Ar^{Tfm} male mice measures mitochondrial respiration using oxygen consumption rate (OCR). **(B)** Quantified measures of basal respiration, maximal respiration, and spare respiratory capacity from panel A. **(C)** Seahorse GlycoStress test on differentiated Th2 cells from WT female, WT male, and Ar^{Tfm} male mice to measure glycolysis using extracellular acidification rate (ECAR). **(D)** Quantified measures of basal glycolysis, glycolytic capacity, and glycolytic reserve from panel C. **(E-F)** Expression of MitoSOX Red and Mitotracker green (MTG) in Th2 cells from WT female, WT male and Ar^{Tfm} male mice (n=3-4 mice per group, representative of 2 independent experiments). **(B-F)** Graphs show mean ± SEM, *p<0.05 or as shown, ns not significant, ANOVA with post-hoc Tukey testing. **(G-H)** EM pictures and quantification of Th17 cells from males and Ar^{Tfm} male mice. Images are representative of 3 biological replicates for each group. Quantification was done on 100-110 mitochondria per sample. *p<0.05 or as shown, ns not significant, unpaired T test.

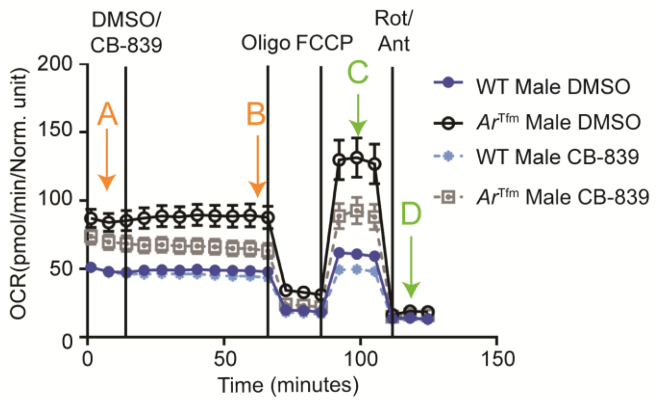

Endpoint	Calculation
Acute Response	ΔOCR: (Last injection before oligomycin, B) – (basal respiration, A)<br><br>ΔΔOCR: $\Delta OCR_{CB\text{-}839} - \Delta OCR_{DMSO}$
Maximal Respiration	Max Respiration: (Max measurement after FCCP, C) – (Minimum measurement after Rot/Ant, D)<br><br>ΔOCR_{max}: $OCR_{MaxCB\text{-}839} - OCR_{MaxDMSO}$

Supplemental Figure 8. Calculations and diagram for Substrate Oxidation Assay. Related to Figure 4. Calculations for Substrate Oxidation Assay.

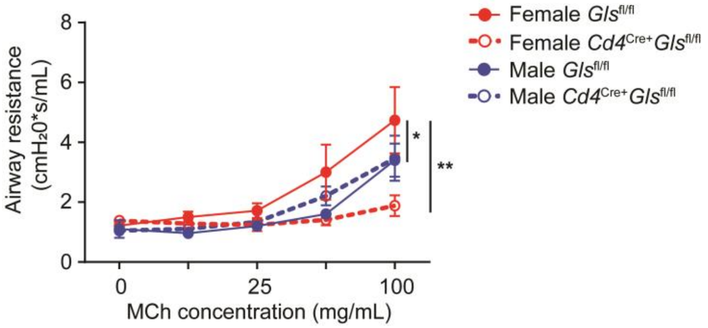

Supplemental Figure 9. Glutaminolysis is required for HDM-induced AHR in female but not male mice. Related to Figure 4. Airway hyperresponsiveness as measured by FlexiVent with increasing doses of methacholine was also conducted on HDM-challenged female and male *Gls*$^{fl/fl}$ and *Cd4*$^{Cre+}$ *Gls*$^{fl/fl}$ mice . n=4-6 mice per group and are representative of two independent experiments, *p<0.05, ** p<0.01, ns not significant, ANOVA of repeated measures with Bonferroni post hoc analysis.

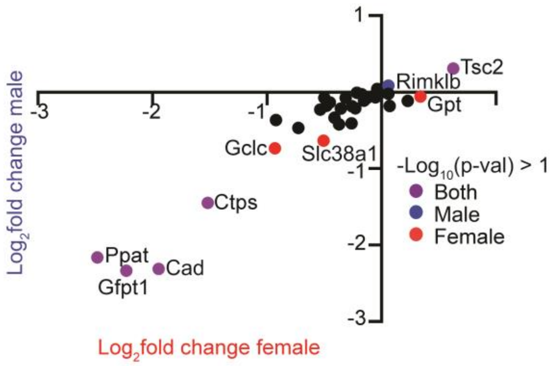

Enzyme	Female -Log$_{10}$(p-value)	Male -Log$_{10}$(p-value)
Gfpt1	3.81	2.70
Ppat	3.84	3.71
Cad	3.20	2.91
Ctps	1.67	2.10
Gclc	2.18	0.95
Slc38a1	1.24	0.32
Gpt	1.84	0.08
Rimklb	0.32	1.84
Tsc2	1.38	1.38

Supplemental Figure 10. AR signaling reduces glutamine uptake in T cells. Related to Figure 5. In vivo CRISPR screen using library targeting glutamine metabolism was conducted using Th17 cells from male and female OT-II Cas9 mice in vivo. Change in gRNA abundance in lung Th17 cells from females and males after OVA-induced lung inflammation with table showing statistics (n=4-5 mice, statistical analysis by MAGeCK, significantly affected represented by color of dot as shown in legend).

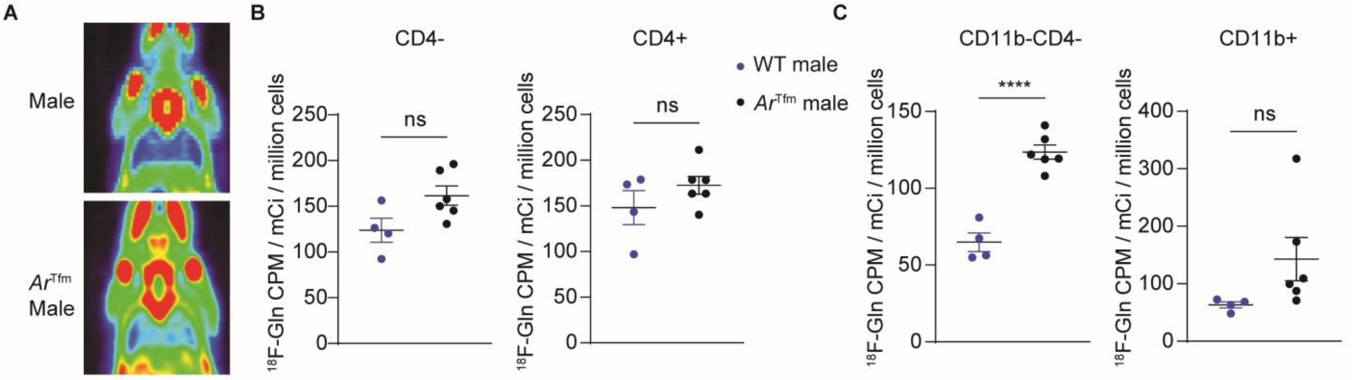

Supplemental Figure 11. AR signaling reduces glutamine uptake in T cells. Related to Figure 5. (A) Images of ^{18}F-glutamine by PET Imaging. **(B)** Quantification of ^{18}F-Glutamine concentration in spleen CD4- and CD4+ cells by magnetic separation and gamma counting, normalized to viable cells for WT male and *Ar*^{Tfm} male mice (n=4-6 mice per group). **(C)** Quantification of ^{18}F-Glutamine concentration in lung CD11b-CD4- cells and CD11b+ cells by magnetic separation and gamma counting, normalized to viable cells (n=4-6 mice per group).

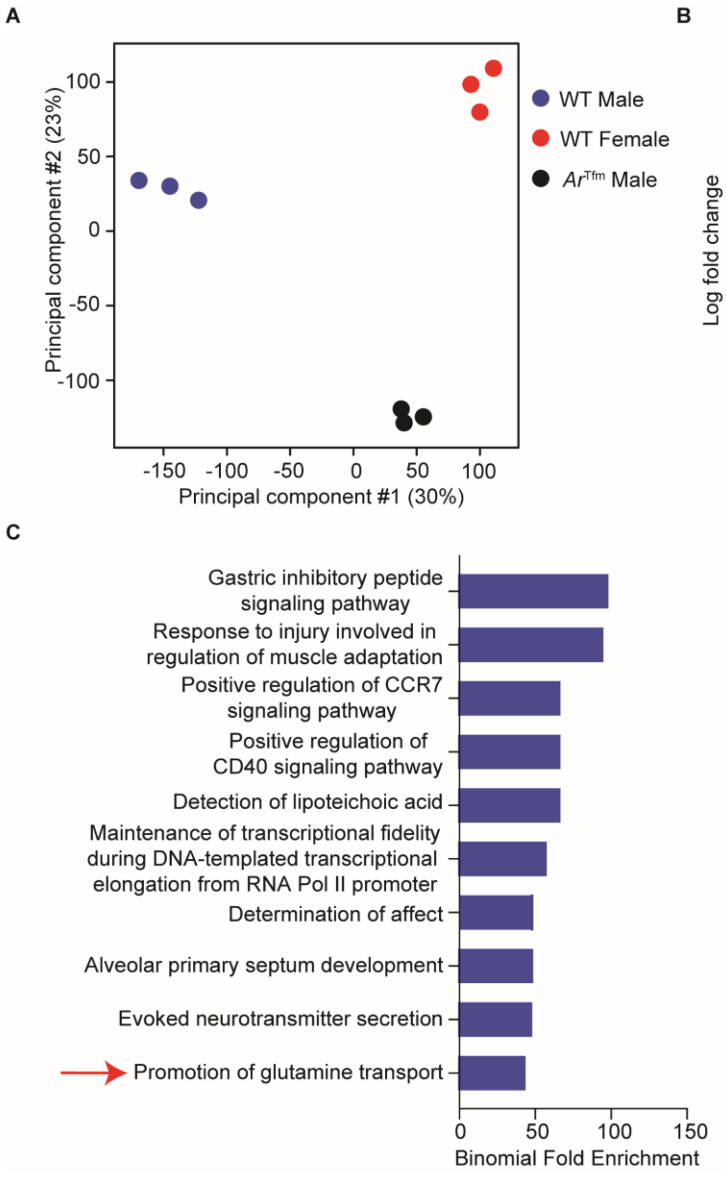

Supplemental Figure 12. AR signaling alters H3K27 trimethylation in Th17 cells. Related to Figure 7. (A) PCA for H3K27me3 analysis of Th17 cells from WT female, WT male, and *Ar*^{Tfm} male mice. **(B)** Analysis of differentially methylated areas in Th17 cells from female and *Ar*^{Tfm} male mice. **(C)** GREAT analysis to find differences in gene ontology pathways in Th17 cells from WT male versus WT female mice. Pathways shown had a region-based binomial FDR q-value <0.05.

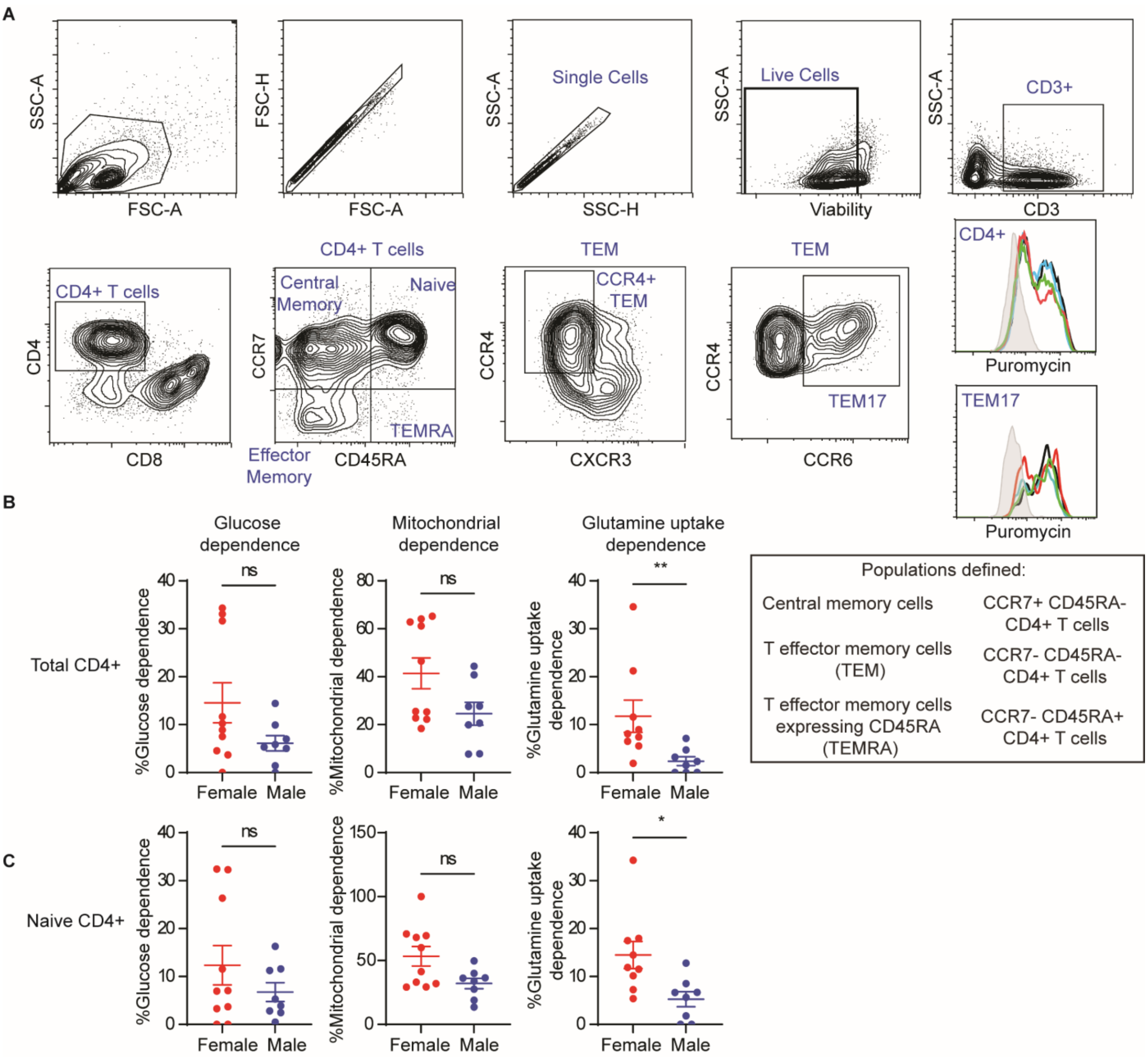

Supplemental Figure 13. Males with severe asthma have decreased dependence upon glutamine uptake in circulating CD4+ T cell subsets compared to females with severe asthma. Related to Figure 8. (A) Gating strategy for SCENITH experiments. **(B-C)** Glucose dependence, mitochondrial dependence, and glutamine uptake dependence in males and females measured using SCENITH inhibitors - 2-deoxyglucose (blue), oligomycin (red), and V9302 (green), all inhibitors (gray), and vehicle (black) in total CD4+ T (defined as Live, CD3+, CD4+, CD8-) and Naive CD4+ T cells (defined as Live, CD3+, CD4+ CD8-, CD45RA+, CCR7+). All graphs show mean ± SEM. *p<0.05, **p<0.01, ns: not significant, or p-value as shown; two-tailed Mann-Whitney U test.

Supplemental Table 1. CYTOF panel. Related to Figure 1 and Figure S1-S2.

Marker	Clone	Mass	Marker	Clone	Mass
CD45	HI30	89	CXCR3	G025H7	156
Dead Rhodium	-	103	CD137	4B4-1	158
CD66b	80H3	108	CCR7	G043H7	159
CD8a	RPA-T8	109	CTLA4	14D3	161
CD16	3G8	110	Glut1	NB110-39113	163
CD14	M5E2	111	CD95	DX2	164
CD4	RPA-T4	112	CD44	BJ18	166
CD19	HIB19	113	CD38	HIT2	167
CD3	UCHT1	116	CYTOC	6H2.B4	168
CD45RO	UCHL1	141	CD25	2A3	169
CPT1a	8F6AE9	142	CD45RA	HI100	170
CD127	A019D5	143	CXCR5	RF8B2	171
ATP5a	7H10BD4F9	144	CD57	HCD57	172
GRIM19	6E1BH7	145	CXCR4	12G5	173
CD20	2H7	147	HLA-DR	L243	174
CD27	L128	148	PD-1	EH12.2H7	175
CCR4	205410	149	CD56	CMSSB	176
CD134	ACT35	150	Cells Iridium	-	191
ICOS	C398.4A	151	Cells Iridium	-	193
TCRγδ	11F2	152	CD11b	ICRF44	209
GLUD1	polyclonal	154			

Supplemental Table 2. Patient demographics for CYTOF on lymph nodes. Related to Figure 1 and Figure S1-S2.

		**Females (n=14)**	**Males (n=17)**
	Age (mean + SD)	37.7 ± 11.3	36.0 ± 11.5
**Race**	African American	5 (35.7%)	4 (23.5%)
	Asian	0 (0%)	1 (5.9%)
	Native American	0 (0%)	0 (0%)
	White	7 (50.0%)	11 (64.7%)
	Other	2 (14.3%)	1 (5.9%)
**Ethnicity**	Non-Hispanic	12 (85.7%)	16 (94.1%)
	Hispanic	2 (14.3%)	1 (5.9%)
**Cause of Death**	Accident	2 (14.3%)	7 (41.1%)
	Cardiac arrest	0 (0%)	1 (5.9%)
	Seizure	0 (0%)	2 (11.8%)
	Stroke	2 (14.3%)	5 (29.4%)
	Unknown	10 (71.4%)	2 (11.8%)

Supplemental Table 3. Metabolic pathway analysis on Th17 cells from Ar^{Tfm} male mice compared to wild-type male mice. Related to Figure 4.

Pathway	FDR-adjusted p-value	Impact
Glycerophospholipid metabolism	0.0102	0.03
Glycine, serine and threonine metabolism	0.0102	0.05
Arginine and proline metabolism	0.0102	0.42
Aminoacyl-tRNA biosynthesis	0.0102	0.00
Histidine metabolism	0.0102	0.22
D-Glutamine and D-glutamate metabolism	0.0102	0.50
Glyoxylate and dicarboxylate metabolism	0.0102	0.00
Nitrogen metabolism	0.0102	0.00
Porphyrin and chlorophyll metabolism	0.0102	0.00
Glutathione metabolism	0.0102	0.05
Arginine biosynthesis	0.0102	0.25
Nicotinate and nicotinamide metabolism	0.0141	0.00

Supplemental Table 4. Patient demographics for SCENITH on PBMCs. Related to Figure 8 and S13.

		**Females (n=10)**	**Males (n=8)**
	Age (mean + SD)	36.5 ± 5	33.1 ± 9.9
**Race**	African American	4 (40.0%)	3 (37.5%)
	Asian	2 (20.0%)	0 (0.0%)
	Native American	0 (0.0%)	0 (0.0%)
	White	2 (20.0%)	4 (50.0%)
	Other	2 (20.0%)	1 (12.5%)
**Ethnicity**	Non-Hispanic	12 (85.7%)	7 (87.5%)
	Hispanic	2 (14.3%)	1 (12.5%)

Supplemental Table 5. Antibodies used in this project.

Antibody	Manufacturer	Catalog number
Anti-Human CD45	Fluidigm	Cat# 3089003
Anti-Human CD66b	BioLegend	Cat# 305102
Anti-Human CD8a	BioLegend	Cat# 301053
Anti-Human CD16	BioLegend	Cat# 302051
Anti-Human CD14	BioLegend	Cat# 301843
Anti-Human CD4	BioLegend	Cat# 300541
Anti-Human CD19	BioLegend	Cat# 302247
Anti-Human CD3	BioLegend	Cat# 300443
Anti-Human CD45RO	BioLegend	Cat# 304239
Anti-Human CPT1a	Abcam	Cat# ab128568
Anti-Human CD127	Fluidigm	Cat# 3143012B
Anti-Human ATP5a	Abcam	Cat# ab110273
Anti-Human GRIM19	Abcam	Cat# ab110240
Anti-Human CD20	Fluidigm	Cat# 3147001B
Anti-Human CD27	BD Custom	Cat# 624084
Anti-Human CCR4	Fluidigm	Cat# 3149029A
Anti-Human CD134	Fluidigm	Cat# 3150023C
Anti-Human ICOS	Fluidigm	Cat# 3151020B
Anti-Human TCRγδ	Fluidigm	Cat# 3152008B
Anti-Human GLUD1	Abcam	Cat# ab34786
Anti-Human CXCR3	Fluidigm	Cat# 3156004B
Anti-Human CD137	Fluidigm	Cat# 3158013B
Anti-Human CCR7	Fluidigm	Cat# 3159003
Anti-Human CTLA-4	Fluidigm	Cat# 3161004B
Anti-Human Glut1	Novus Biologicals	Cat# NB110-39113
Anti-Human CD95	Fluidigm	Cat# 3164008B
Anti-Human CD44	Fluidigm	Cat# 3166001C
Anti-Human CD38	Fluidigm	Cat# 3167001B
Anti-Human CYTOC	BD Biosciences	Cat# 556432
Anti-Human CD25	Fluidigm	Cat# 3169003
Anti-Human CD45RA	Fluidigm	Cat# 3170010
Anti-Human CXCR5	Fluidigm	Cat# 3171014B
Anti-Human CD57	Fluidigm	Cat# 3172009B
Anti-Human CXCR4	Fluidigm	Cat# 3173001B
Anti-Human HLA-DR	Fluidigm	Cat# 3174001
Anti-Human PD-1	Fluidigm	Cat# 3175008
Anti-Human CD56	Fluidigm	Cat# 3176003B
Anti-Human CD11b	Fluidigm	Cat# 3209003
Anti-Mouse CD45 BV786	BD Biosciences	Cat# 564225
Anti-Mouse CD45 Nova Fluor Blue 610-70S	Thermo Fisher Scientific	Cat# M005T02B06
Anti-Mouse CD3 APC-Cy7	BioLegend	Cat# 100222
Anti-Mouse CD3 Pacific Blue	BioLegend	Cat# 100334
Anti-Mouse CD4 Alexa Fluor 700	BioLegend	Cat# 100429
Anti-Mouse CD4 PE-Cy5	BD Biosciences	Cat# 553654
Anti-Mouse IL-13 PE-Cy7	Thermo Fisher Scientific	Cat# 25-7133-82

Anti-Mouse IL-17A BV605	BD Biosciences	Cat# 564169
Anti-Human/Mouse Gata3 PE-CF594	BD Biosciences	Cat# 563510
Anti-Mouse RORγT BV786	BD Biosciences	Cat# 564723
Anti-Human Recombinant GLUD1+GLUD2 Alexa Fluor 488	Abcam	Cat# ab204001
Anti-Mouse phospho-S6 Alexa Fluor 700	Cell Signaling Technology	Cat# 27036
Anti-Human/Mouse Glut1 Alexa Fluor 647	Abcam	Cat# ab195020
Anti-Human/Mouse HIF-1α	R&D Systems	Cat# IC1935P
Anti-Mouse CD3e	BD Biosciences	Cat# 553057
Anti-Mouse CD28	BD Biosciences	Cat# 553295
Anti-Mouse IFNγ	BioLegend	Cat# 505847
Anti-Mouse IL-4	BioLegend	Cat# 504122
Anti-Human/Mouse Tri-Methyl-Histone H3 (Lys27)	Cell Signaling Technology	Cat# 9733S
Anti-Human CD3 Alexa Fluor 700	BioLegend	Cat# 317339
Anti-Human CD4 BV510	BioLegend	Cat# 317444
Anti-Human CD8α BV570	BioLegend	Cat# 301037
Anti-Human CCR7 BV650	BioLegend	Cat# 353233
Anti-Human CD45RA APC-Vio 770	Miltenyi Biotec	Cat# 130-113-925
Anti-Human CXCR3 PE-Cy5	BioLegend	Cat# 353755
Anti-Human CCR4 PE-Vio 770	Miltenyi Biotec	Cat# 130-118-496
Anti-Human CCR6 APC	BioLegend	Cat# 353416
Anti-Puromycin Alexa Fluor 488	Millipore Sigma	Cat# MABE343-AF488

Supplemental Table 6. $^{13}C_5$-Glutamine SIL Species Analyte parameters. Related to Figure 6.

$^{13}C_5$-Glutamine SIL Species Analyte	Q1 (m/z)	Q3 (m/z)	RT (min)
$^{13}C_3$-Pyruvate	90	45	3.1
$^{13}C_1$-Lactate (C1) [INTERNAL STANDARD]	90	43	5.5
$^{13}C_2$-Acetyl-CoA	810	426	13.0
$^{13}C_2$-Citrate/Isocitrate	193	113	13.4
$^{13}C_4$-Citrate/Isocitrate	195	114	13.4
$^{13}C_5$-Citrate/Isocitrate	196	116	13.4
$^{13}C_6$-Citrate/Isocitrate	197	116	13.4
$^{13}C_2$-*cis*-Aconitate	175	86	12.5
$^{13}C_4$-*cis*-Aconitate	177	88	12.5
$^{13}C_5$-*cis*-Aconitate	178	89	12.5
$^{13}C_6$-*cis*-Aconitate	179	89	12.5
$^{13}C_3$-a-Ketoglutarate	148	104	10.3
$^{13}C_5$-a-Ketoglutarate	150	105	10.3
$^{13}C_2$-Succinyl-CoA	868	426	13.5
$^{13}C_3$-Succinyl-CoA	869	426	13.5
$^{13}C_4$-Succinyl-CoA	870	426	13.5
$^{13}C_2$-Fumarate	117	73	11.0
$^{13}C_3$-Fumarate	118	73	11.0
$^{13}C_4$-Fumarate	119	74	11.0
$^{13}C_2$-Malate	135	117	11.4
$^{13}C_3$-Malate	136	118	11.4
$^{13}C_4$-Malate	137	119	11.4
$^{13}C_2$-Oxaloacetate	133	89	4.8
$^{13}C_3$-Oxaloacetate	134	90	4.8
$^{13}C_4$-Oxaloacetate	135	90	4.8
$^{13}C_3$-Glutamine	148	130	9.5
$^{13}C_5$-Glutamine	150	132	9.5
$^{13}C_3$-Glutamate	149	131	11
$^{13}C_5$-Glutamate	151	133	11
$^{13}C_2$-Aspartate	134	89	11.2
$^{13}C_3$-Aspartate	135	90	11.2
$^{13}C_4$-Aspartate	136	91	11.2
$^{13}C_3$-GSH	309	128	11.1
$^{13}C_5$-GSH	311	128	11.1
$^{13}C_6$-GSSG	617	309	13.7
$^{13}C_{10}$-GSSG	621	311	13.7

Q1 and Q3: mass charge ratio (m/z) for precursor and product ions; RT – retention time
